# Localisation Microscopy of Breast Epithelial ErbB-2 Receptors and Gap Junctions: Trafficking after γ-Irradiation, Neuregulin-1β, and Trastuzumab Application

**DOI:** 10.3390/ijms18020362

**Published:** 2017-02-09

**Authors:** Götz Pilarczyk, Ines Nesnidal, Manuel Gunkel, Margund Bach, Felix Bestvater, Michael Hausmann

**Affiliations:** 1 Kirchhoff Institute for Physics, Im Neuenheimer Feld INF 227, D-69120 Heidelberg, Germany; ines.nesnidal@kip.uni-heidelberg.de (I.N.); margund.bach@kip.uni-heidelberg.de (M.B.); hausmann@kip.uni-heidelberg.de (M.H.); 2 Bioquant-University of Heidelberg, Im Neuenheimer Feld INF 267, D-69120 Heidelberg, Germany; manuel.gunkel@bioquant.uni-heidelberg.de; 3 German Cancer Research Center, Im Neuenheimer Feld INF 280, D-69120 Heidelberg, Germany; f.bestvater@dkfz.de

**Keywords:** breast cancer, ErbB receptor tyrosin kinase, gap junction, retrograde trafficking, irradiation, DNA injury, neuregulin, therapeutic antibody, spectral precision distance microscopy

## Abstract

In cancer, vulnerable breast epithelium malignance tendency correlates with number and activation of ErbB receptor tyrosine kinases. In the presented work, we observe ErbB receptors activated by irradiation-induced DNA injury or neuregulin-1β application, or alternatively, attenuated by a therapeutic antibody using high resolution fluorescence localization microscopy. The gap junction turnover coinciding with ErbB receptor activation and co-transport is simultaneously recorded. DNA injury caused by 4 Gray of 6 MeV photon γ-irradiation or alternatively neuregulin-1β application mobilized ErbB receptors in a nucleograde fashion—a process attenuated by trastuzumab antibody application. This was accompanied by increased receptor density, indicating packing into transport units. Factors mobilizing ErbB receptors also mobilized plasma membrane resident gap junction channels. The time course of ErbB receptor activation and gap junction mobilization recapitulates the time course of non-homologous end-joining DNA repair. We explain our findings under terms of DNA injury-induced membrane receptor tyrosine kinase activation and retrograde trafficking. In addition, we interpret the phenomenon of retrograde co-trafficking of gap junction connexons stimulated by ErbB receptor activation.

## 1. Introduction

The presented matter focuses on ErbB receptor protein molecules, either as isolated residues in the cell plasma membrane or in the form of dimers and oligomeres spanning the plasma membrane or being packed into intracellular carriers. Further described are connexin-43 transmembrane proteins, as constituents of the plasma membrane-residing connexons and gap junctions. After stimulation of ErbB receptors by ionizing irradiation or neuregulin-1 (NRG-1) application or receptor attenuation by application of the therapeutic antibody trastuzumab (also called herceptin under commercial prospects), a combined microscopy study using confocal laser scanning microscopy (CLSM) and localization microscopy (LM) reveals the dynamics of protein accumulation and trafficking.

The mamma carcinoma is the predominant form of cancer in females in postindustrial societies. The individual lifetime incidence probability ranges at 13%, with a sub-ratio of 5%–15% of these cases being males [1]—equivalent to below 1% total. An increased copy number or transcription of the ErbB gene (Her2/neu) results in an abnormally high receptor density in malignant breast tissue [2] and in bad patient prognosis [3]. In breast cancer, the ErbB-2/ErbB-3 dimer is the main combination responsible for receptor activity [4]. This activation is accompanied by an increase in the density of exclusively activated ErbB-2 receptors, further by the inclusion into membrane-coated transport vesicles and by the nucleograde transport across the cytosol. To visualize structural alterations during receptor mobilization, the nanometer regime for receptor aggregates and the μm scale for cytosolic transport have to be observed.

The mentioned packing and mobilization activity can function as a tag for malignant transition in breast epithelium. Several parameters of both functional and structural character influence ErbB dimer receptor tyrosine kinases (RTK) activity. During the onset of malignancy, cells of the mammal epithelium convert their cell homeostatic character into proliferation activity [5]. The so-called epithelial–mesenchymal transition includes orchestrated activation of cytosolic phosphorylation cascades involving protein kinase C (PKC) and Wnt dependent kinases [6]. The protein kinases AKT (also called PKB) and PKC are substrates of dimerized ErbB receptor RTKs [7]. Consequently, the ErbB receptor-mediated cytosolic phosphorylation activity is a reporter for the induction of malignant transformation in breast epithelium, and blocking ErbB activity acts supportive in breast cancer chemotherapy [8]. This can be recorded by correlative microscopy covering the μm as well as the nm cytosol regime.

Disturbed receptor protein turnover by insufficient retrograde receptor trafficking increases the number of ErbB receptors in genetically-normal cells [9]. The activity of the ErbB receptor dimers is extended when they are stabilized by chaperone binding in the cytosol [10]. The RTK activity of ErbB receptor dimers is accompanied by a retrograde trafficking with the perinuclear cytosol [11] or the nucleus interior [12] being targets addressed. Once in the nuclear matrix, the ErbB receptor kinase function on histone H4 tyrosine side chains is involved in regulation of DNA repair processes [13,14]. This process of activation-induced receptor sequestration is a parameter accessible by combined conventional microscopy and nanoscopy.

The mobilization process of activated ErbB receptors towards their nuclear region of activity is a process that is easy to follow by conventional microscopy. Besides a direct activation of ErbB receptors through the extracellular dimerizing peptide neuregulin-1β [15,16], DNA injury induced by γ-irradiation results in caveolin-1 supported nucleograde trafficking of activated ErbB receptor dimers towards sites of DNA repair [17,18]. Hereby, the nuclear pore protein Nup358 and importin-1β interact to facilitate the passage of ErbB receptor across the nucleopore into the nuclear matrix. This event releases the ErbB receptors from the cytosolic transport vesicles, which have been generated at the plasma membrane and transported across the cytosol [19]. Vesicle formation and receptor packaging generate transport cytosolic elements, their dimensions and dynamics closely fitting the spatial and temporal resolution capacity of localization microscopes.

Double-strand breaks in the densely-packed DNA nucleosome complexes locally release the broken DNA strands from their nucleosomal carriers [20,21]. The process accumulates both free DNA fibers, and in a regulated fashion, liberated structural and regulative histones and other regulator proteins [22]. Among the released proteins are chaperones, also called heat shock proteins (HSPs) [23,24,25], and actin related proteins (ARPs) [26,27]. Both protein families are involved in the repair of injured DNA in the nucleus [28] and in the activation of receptor tyrosine kinases, and thus in the activation of downstream associated phosphoinositol trisphosphate kinase (PI3K) and serine/threonine specific kinases (Akt) kinase pathways [29]. In the chaperone protein family, the heat shock protein 90 subtype α (HSP90α) is of particular interest due to its prominent function in breast cancer development and as a therapy target [30]. HSP90α is accumulated in nuclear DNA repair foci, where it is phosphorylated by the DNA repair complex named DNA-dependent protein kinase, catalytic subunit (DNA-PKs) [31,32]. In addition, HSP90α associates with ErbB receptor dimers and is active in retrograde trafficking of this receptor tyrosine kinases towards the nucleoplasm [9]. By the mechanism of DNA injury-stimulated HSP90α activation and ErbB receptor nucleograde trafficking with subsequent ErbB kinase activity in the nucleus, a closed loop between DNA repair and ErbB mobilization establishes. The resulting DNA repair processes can be followed by localization microscopy.

In addition to the described canonical feedback loops between injured nuclear DNA and ErbB receptor dimers, a side activity of chaperones and actin-related proteins affects gap junction turnover. Gap junctions are tetrameric transmembrane protein complexes organized in groups with sixfold symmetry called connexons and arranged in crystal-approximating 2D arrays. Two opposing arrays in neighboring cells face one another and constitute a field of channel hexameres called a gap junction [33]. While being a main structure for electrical potential spread in cardiac myocytes, gap junctions in epithelial cells regulate inter-cellular ions and pH homeostasis, and are regulators of isotonic state and cytosolic pressure [34,35,36]. Gap junctions are further targets for extracellular peptides like neuregulin-1β [37], and influence the cytosolic access for chemotherapeutics [38,39]. Regardless of molecular weight and geometric size [40], complex production in ER and Golgi apparatus [41,42,43], and precise spatial deposition by the actin cytosceleton and microtubules [44,45], gap junctions are of high mobility in the plasma membrane and are subjected to a rapid turnover with half-value periods of around few hours [46]. This flexibility and turnover makes them promising targets for the precise regulation of activity and movement by cell physiological interactors [47]. Consequently, gap junctions are not only subject to anterograde and retrograde trafficking, but are also the substrate of several protein kinases [48,49] influencing channel permeability and stability. In the gap junction protein connexin 43 (Con 43), a cytosolic loop is the target for several protein kinases and carries protein binding motives of the spectrin homology (SH) type [50]

Activated ErbB receptor dimers phosphorylate the downstream protein kinases PKA, PKB (also called AKT), and PKC [51,52], and induce further phosphorylation of serine residues on the cytoplasmic loop of the gap junction protein connexin 43 [53]. The thus activated kinases target proteins which regulate the physical contact between the subcortical f-actin cytoskeleton and plasma membrane-located gap junctions [48]. Further, gap junction precursors under anterograde trafficking are affected, because the spatial interaction between microtubule capping structures and gap junction components is a common target for protein kinases [54,55]. Both interactions modulate the sorting of transported and membrane-residing gap junctions [56]. In microscopy evaluation, this particular receptor load transported can be observed directly.

The mobilization of ErbB receptor dimers and gap junctions interfere in several ways. Nuclear DNA injury releases signaling factors like HSPs and ARPs [28,57] which target not only Erb receptor dimers and induce caveolin and importin-β dependent nucleograde transport [58,59], but also affect the retrograde trafficking of gap junctions [60,61,62]. This initiates transport routes which guide internalized and vesicle-packed gap junctions towards the perinuclear cytosol by utilizing structural, functional, and regulation components of the regular retrograde trafficking machinery. The retrograde trafficking involves the sequestration of gap junctions in clathrin-coated pits and vesicles. The thus-generated transport units are decorated with β-importin. This process is a close imitation of the ErbB receptor nucleograde trafficking [19], but in case of gap junction transport, the interaction between HSPs and nuclear pore proteins (NUPs) fails in total or does not mediate the protein carriage across the nuclear pore complexes [63,64,65] into the nuclear matrix, as in the case of ErbB receptor dimers. By microscopy examination, an increase in gap junction packing density accompanies a nucleograde dislocation.

The coordinated physiological activity and the temporally and spatially synchronized mobilization of ErbB receptor family members and gap junction protein complexes is a key factor in carcinogenesis induced by intrinsic genetic transformations and by extrinsic mutagen factors like high energy irradiation, the epithelial-to-mesenchymal transition, or the presence of carcinogenic compounds [66,67,68]. Further processes touched by this cell activity are the generation of cancer stem cells and the loss of growth inhibitory signal perception during carcinogenesis and intercellular communication [69,70,71]. This makes the synchronized regulation of ErbB receptors and gap junctions a promising concern in tumor diagnosis, and focuses on gap junctions as a target for chemotherapy [72,73,74,75].

In conclusion, it can be stated that dimerized ErbB receptors start cytosolic kinase cascades having the gap junctions’ cytosolic regulator moieties as targets, and share common features of retrograde respectively nucleograde trafficking [76]. This is an input value for the analysis of crossed-over regulation loops between injured DNA-induced ErbB receptor mobilization, neuregulin-1β-induced combined mobilization of ErbB receptor dimers and gap junctions, and the mobilization of gap junctions by DNA-injuring irradiation without agonist-mediated ErbB receptor activation.

For the analysis of the processes sketched above, a combined examination approach using confocal laser scanning microscopy (CLSM) and a microscopy method facilitating optical identification of objects in the protein assembly size regime is a promising choice [77,78]. The focus on the overall cytosolic distribution dynamics by the use of CLSM on one hand and the precise evaluation of receptor protein ensemble density distributions by distance distribution histograms [79] on the other hand makes it possible to compare receptor packing and receptor localization after stimulus application [80]. Super-resolution microscopy-based approaches are exceedingly promising, because it is possible to derive spatial information comparable to electron microscopy imaging but in a cell system very closely resembling the native state [81]. No other method is capable of covering a magnification range by a factor of 10${}^{4}$ in a fully-hydrated cell specimen which is probed not only for spatial protein distribution, but simultaneously for functional interactions.

## 2. Results

As suggested for ErbB receptors, connexin-43 protein molecules can also be found at different levels of organization. An increase of this level as accompanying the transition from connexons to gap junctions [82] faces a decrease of organization level when the connexon is subjected to anterograde or retrograde trafficking [83]. For both proteins, organization levels are associated with physiological activity and subcellular accumulation [51,84,85]. As a consequence, an experimental approach combining a controlled stimulation of ErbB receptor and gap junction physiological activity with a close observation of the mentioned proteins by microscopy will greatly benefit from addressing the discrete appearance of the proteins as individual molecules, but also mentioning the protein ensembles as dense and continuous protein populations at several intracellular locations [79].

Such a combination of two different microscopy approaches accounts for the discrete and the continuous nature of ErbB receptor and gap junction residues. It takes into consideration that the ErbB receptor and connexin-43 proteins are isolated molecules a priori, but interact, and this way organize well a defined continuum de facto. However, the individual properties and restrictions of both microscopy approaches must be carefully taken into account [86,87,88]. Therefore, a microscopy system evaluation with special regard to the biological specimen must be used to start specification and interpretation of the cell biology results. Figure S1 given in supplementary materials gives a survey of both microscopy methods, starting with induction and handling of fluorescence signals and finishing with the display of protein densities and distributions inside the specimen.

### 2.1. ErbB Receptor and Connexin-43 Protein Visualization and Measurement by CLSM and LM

ErbB receptor protein molecules and connexin-43-containing gap junction complexes are occupied by a dynamical set of accumulation, dissociation, and higher-order organization processes. During the transition between these types of ensembles, the characteristic molecule density distributions are altered dramatically.

The distance data processing and diagram display can be interpreted in a simple semi-quantitative way (Figure S1h1–h3). There are three types of image signal distribution of special interest for ErbB receptor and connexin-43 protein localization in mamma-epithelial cells. In Figure S1h1, the distances between randomly distributed proteins are evaluated using a Ripleys K/L method and displayed as amount of protein distances over the corresponding distance value. With the red dot in the coordinate origin as a common center of a series of circles with increasing radius values, collecting a continuously increasing number of protein molecules is charted by the increasing radial distance. As a consequence, the distance diagram is of the form of an increasing line for random protein distribution.

The same data evaluation process gives different results if applied to receptor molecules organized in several clusters (Figure S1h2). Inside every separated cluster, the individual protein molecules distribute over a characteristic set of distances. This results in peak accumulation in the density diagram. In case the cluster is not symmetrically distributed around the center of evaluation (the red asterisk), the distance between the center of evaluation and the center of the cluster manifests in the center value of the peak. This value does not need to meet the peak maximum value if the cluster does not follow a symmetry primitive. The width of the peak reflects variations in protein distances inside the cluster. This is not identical to the absolute cluster density or cluster size, but it must be regarded that a small cluster is not capable of giving space and particles for a high number of density variations.

Figure S1h3 summarizes a set of typical situations in ErbB receptor and connexon/gap junction clustering. The cases L1 (blue) and L3 (green) represent situations with a non-primitive cluster shape of low (green) and higher (blue) protein molecule densities not eccentric with respect to the origin of evaluation (asterisk). The molecule distribution inside the cluster is random, resulting in a symmetric peak appearance. In contrast, situation L2 (black) shows a cluster with an additional level of organization. The black crosses fall into groups of four individuals. The groups themselves are spatially discriminated from each other. The small-scale organization level results in a sharp peak at low distance values, while the superposed group distances produce a second peak at higher diagram values.

The repetition of image recordings at one specimen location progressively closes the gaps between the isolated fluorescence signals without superposition of neighboring signals’ point spreads. This principle does not result in a coherent image of a specimen, but either in an image point cloud with discontinuous character recapitulating the dye distribution in the specimen or in a point matrix with individual coordinate pairs, each pointing to a single fluorescence signal locality. Here, the matrix information can be used to reconstruct a pointillism image or to graphically display point-to-point distances in the form of Cartesian diagrams and histograms, in polar diagrams, or in Voronoi patterns.

Among the plethora of applications using photo-cycle burst localization microscopy, there are several approaches that deal with the interaction of isolated receptor protein molecules in the plasma membrane, as opposed to receptors organized in clusters [89,90,91]. The examinations touch the receptor clustering as a challenge for microscopic density recording dynamics, which is addressed by a receptor titration approach [92]. Approaches based on photoactivated light microscopy (PALM) as well as stochastic optical reconstruction microscopy (STORM) related techniques focus on the clustering dynamics of membrane receptors in cells of the immune system upon stimulation [93,94,95]. Nano-cluster dynamics are identified as a main characteristic of membrane organization, and in addition to the evaluation of particular receptor clusters, the lipid raft environment is also included in observations. The concept is extended to a regulation model for the T-cell receptor (TCR) andits activity at the so-called immunological synapse [96,97,98]. In addition to the examination of membrane proteins, structures in the cytosol and the nucleus have been under focus using localization microscopy approaches with conventional fluorescent markers [99,100,101].

### 2.2. ErbB Receptor Membrane Clustering Modified by NRG-1 or Trastuzumab as Compared to Unstimulated Situation

Receptor clustering covers a receptor spatial distance range in the nanometer regime, ranging from some nanometers in the case of the dimers to some hundred nanometers for the cluster diameter. This indicates that even the size of the whole cluster aggregate is not accessible by confocal laser scanning microscopy (CLSM). A realistic assumption for the lateral CLSM resolution being 400–500 nm will designate any ErbB receptor pattern in the CLSM images as coming from large range organization and dynamics not related to basic clusters. Nonetheless, ErbB receptor accumulations appear as reacting to the application of neuregulin-1 (NRG-1), trastuzumab, and irradiation. Such a reaction is supported by the literature, where the stimulation of ErbB receptors with NRG-1, the attenuation of ErbB activity by the therapeutic antibodies against ErbB 2 or ErbB 3 receptors, and the impact of high-energy irradiation is reported [10]. A displacement of ErbB receptors over distances in the whole cell diameter regime can be induced [9].

In difference, the localization microscopy (LM)-derived localization matrix identifies receptor dynamics related to the primary receptor cluster population. The distance range between 5 and 250 nm cover the individual ErbB receptor molecules as well as the clusters.

**Figure 1:**
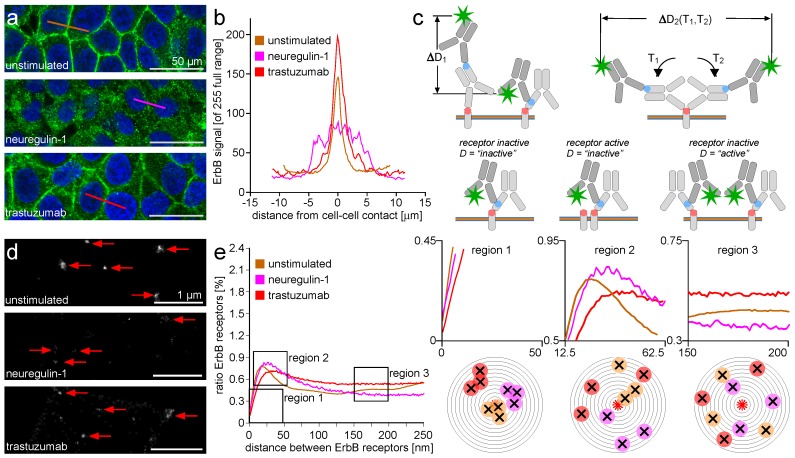
ErbB-2 receptor nm range organization and μm range movement upon activation by neuregulin-1 (NRG-1) and attenuation by the therapeutic antibody trastuzumab. Illustration of the short-range distance deterioration after ErbB-2 by secondary antibody identification and the resulting distance distribution spectra. (**a**) A nearly confluent monolayer of MCF-7 mamma-epithelial cells recorded by confocal laser scanning microscopy (CLSM). The cells are fixed and stained for ErbB-2 receptors by indirect immunofluorescence (green) and for the cell nucleus by the nucleic acid sensitive stain using 4${}^{\prime }$,6-diamidino-2-phenylindole (DAPI) (blue). Comparison of an unstimulated monolayer (top) with a monolayer after NRG-1 stimulation (middle) and trastuzumab attenuation (bottom). The overall receptor distribution appears accumulated at the cell-to-cell contacts for the unstimulated and attenuated cells and mobilized into the cytosol NRG-1-mediated cell stimulation. The golden, purple, and red lines indicate a typical cell location for the generation of intensity line plots. (**b**) Intensity line plots for for the regions of interest (ROIs) selected in the cell monolayer for unstimulated (golden trace), NRG-1 stimulated (purple trace), and trastuzumab attenuated (red trace) cells. The abscissa origin (0) is normalized to the cell-to-cell contact. (**c**) A set of alternative typical spatial orientations of the primary to secondary antibody arrangement after indirect immuno-staining. The plasma membrane is represented in side view by the triple layered with the orange center and the blue border. The round corner box with the red dot represents the ErbB receptor and the epitope for the primary antibody (light grey). The primary antibody itself presents an epitope for the binding of the secondary antibody (dark grey). Variations in the spatial orientation of this group is responsible for the different distances between the ErbB receptor and the fluorescent dye (green asterisk). Alternations inside this set may account for a staining-dependent limit for localization precision. Some varieties of the spatial constellation designate an (activated) receptor accumulate as inactive and vice versa. (**d**) Localization matrix graphical display of the specimen described above but analyzed by localization microscopy (LM). The red arrows hint to local ErbB-2 receptor accumulations. All localities are in direct neighborhood to cell-to-cell contacts. (**e**) Receptor density spectra of the same localization matrix data set with abscissa ranging from 0–250 nm and the ordinate showing the relative ratio (in percent) of the total receptor number. The left diagram displays a complete survey, while three smaller diagrams on the right show typical distance distributions in the survey. The concentric ring series under each small coordinate system gives a graphical interpretation of the particular distance distribution inspired by Ripley’s K/L-based analysis. in each case the red asterisk indicates the common point of the distance measurements. The crosses around the center indicate the loci of protein molecules their distance is to be measured. A ring series acts as a scaffold for the distance classification of the distances between the asterisk in the center and each molecule around. The data set reflecting the center-to-molecule distances can be converted into a data set reflecting the distances between the protein molecules. See text for closer interpretation.

It may be stated that the combined evaluation of CLSM and LM offers the possibility to connect the long-range receptor mobility with the short-range packing density and the mid-range clustering behavior. However, the situation is further complicated by the means of cell epitope identification. The use of monoclonal antibodies against an amino acid sequence in the ErbB receptor protein facilitates the precise identification of this protein in the cell plasma membrane and the cytosol. A second serum-derived antibody carrying several fluorescent dye molecules (typically 5–10) binds to an arbitrary amino acid (AA) sequence in the monoclonal antibody. The population of serum antibodies is heterogeneous with respect to the target epitope bound and also with respect to the locations of the dye molecules in the antibody protein. This implies that a series of spatially- and temporally-overlapping fluorescence emission bursts can originate from one primary/secondary antibody pair. At last, the primary antibody → secondary antibody → fluorescent dye molecule complex can swing over and rotate around the ErbB epitope while randomly emitting fluorescence bursts. These basic conditions of indirect immune identification must be regarded when interpreting the short-range and mid-range dynamics of ErbB receptors after stimulation and attenuation. Figure 1c illustrates the described situation.

Figure 1a shows MCF-7 epithelial cells derived from breast tissue. The ErbB-2 receptors are trans-membrane proteins in the plasma membrane of mamma-epithelial cells [102]. Besides the active form of the receptor located as mentioned above, there are proteins in the state of synthesis embedded in the membrane of the rough endoplasmic reticulum and the Golgi apparatus. In addition, the retrograde transport of receptors included in cytosolic vesicles is reported upon stimulation [7].

CLSM’s typically-increased axial (i.e., perpendicular to the cell monolayer plane) discrimination capacity as compared to wide field microscopy places an optical section across these cells. As a consequence, the out of focus plasma membrane component is not shown, and the cell border appears as a polygonal ring. This makes it possible to discriminate the population of ErbB-2 receptors located in the plasma membrane (i.e., in the ring) from the population in the cytosol (i.e., the area enclosed by the ring). The mentioned ring structure is consolidated in unstimulated and trastuzumab-attenuated MCF-7 cells. The ring vanishes after NRG-1 stimulation, and the number of vesicles in the cytosol increases. It can be deduced that the stimulation by NRG-1 mobilizes the former plasma membrane-residing ErbB-2 receptor population into the cell’s interior. The mobilization process is not induced by trastuzumab application, suggesting that trastuzumab and NRG-1 act in an antagonistic fashion on the ErbB-2 receptor mobility [103]. The effects described can also be seen in the intensity line plots given Figure 1b. The traces show the initial (golden) cell-to-cell contact accumulation vanishing after NRG-1 stimulation (purple), and it is even more pronounced after trastuzumab attenuation (red) of the cells. It is reported that upon breast epithelial cell stimulation by NRG-1, ErbB-2/3 receptor dimers consolidate and develop a cytosolic tyrosine kinase activity [104]. This process is depressed by trastuzumab. It is also reported that ErbB-2/3 dimers are subjected to membrane budding and vesicle formation [11]. The resulting vesicles are transported in a nucleograde fashion, and accumulate in the cytosol around the cell nucleus [19]. This process takes approximately 45 min—a time window too short for the production of new ErbB-2 protein in the rough endoplasmic reticulum. Thus, receptor transport is the main reason for plasma membrane depletion and cytosol receptor accumulation.

The CLSM recording is not vulnerable against the spatial superposition of ErbB-2 receptor location and antibody association to this location, because the CLSM spatial resolution is limited to a value above the spatial size regime where the superposition process happens. This is not the case for LM imaging, and consequently, the loss of localization precision due to indirect antibody staining requires attention. Figure 1c gives a semi-quantitative impression of the situation. The membrane-spanning ErbB-2 receptor is given by the rounded corner light grey box perpendicularly orientated to the plasma membrane or vesicle membranes with are drawn as triple layered objects with two green outer layers including an orange inner layer. The antibody epitope is assumed to be extracellular, and is marked by the orange dot. The primary antibody contacts the ErbB-2 epitope with its antigen binding domain. The relative size of ErbB-2 and antibody approximate real conditions. The ErbB-2 receptor is a small protein of 138 kDa with a substantial part facing the cell interior [105]. An antibody of the IgG type is of a molecular weight of 150 kDa, and has a typical Y-shape with two binding moieties" at the two branched termini [105].

In Figure 1d, the LM recording with subsequent visualization of localization matrix events results in an image of the ErbB-2 clustering in the plasma membrane near the cell-to-cell contact border. At first glance, a simple finding is impressive: the receptor clustering and differences in the cluster formation after stimulation or attenuation are accessible by visual inspection. Red arrows point to ErbB-2 accumulation. The appearance of the accumulations changes if NRG-1 provokes the ErbB-2/3 dimer formation or if the application of trastuzumab suppresses the dimer setup. In the preceding text, it was not possible to have a simultaneous look at both the spatial and the functional aspects of receptor stimulation. Here we can immediately recognize that effector/receptor interaction manifests in an altered molecular appearance.

A direct comparison of unstimulated and NRG-1-stimulated ErbB-2/3 dimer clusters reveals two main properties of the transition. First, the absolute amount of plasma membrane resident ErbB-2 epitopes is decreased, as can be seen by the reduced fluorescence signal level when comparing top image and middle image. This is in accordance with the finding in the CLSM records showing the ErbB-2/3 receptors mobilized into the cytosol. The mobilized receptor fraction is lacking at the plasma membrane. This gives rise to the assumption that the activation state of a NRG-1 stimulated MCF-7 cell is not related to an increased ErbB receptor amount in the plasma membrane. Cell activation is related to receptor mobilization. Second, the plasma membrane clusters appear less dense after NRG-1 application as compared to the NRG-1-depleted situation. Again, the reduced absolute amount of ErbB-2 protein might be the reason, but it must be stated that the reduction of protein might also result in clusters of reduced size but unchanged densities. This is not the case. Cluster diameters remain unchanged, but cluster protein density decreases.

After trastuzumab application, the plasma membrane-located ErbB-2 receptor situation at cell-to-cell contact regions consolidates as compared to the unstimulated case. This recapitulates the CLSM findings where a clear accumulation at the cell borders can also be seen. The LM images show the clusters being more solid than after NRG-1 stimulation, and of a size comparable to clusters in unstimulated cells. It might be deduced that trastuzumab stabilizes the unstimulated situation. This is in close agreement with reports about trastuzumab as an anti-cancer therapeutic agent. Trastuzumab is an antagonist of the action of NRG-1 on MCF-7 cells [106].

The above findings as described by visual image analysis are recapitulated in the density/distance plots shown in Figure 1e. For higher clarity, the regions of the plot that display critical information are extracted from the survey plot and are shown on the right. The left extract plot (region 1) gives an impression about the indirect immuno-stain-induced spatial restriction for recording low distances below 25 nm. In the mentioned size domain, the traces fall together, even if they are different at larger distances (middle and right plot). The analysis scheme under the plot gives an impression of the receptor density distribution subject to distance analysis. The individual receptor locations are very close and overlap, giving rise to the antibody-induced false location effects described above. In the survey plot and in the plot detail at region 2, it is shown that the pronounced distance values inside the clusters are altered by NRG-1 (purple) and trastuzumab (red) as compared to the unstimulated situation (golden). After NRG-1 application, the accumulation of receptor distances shifts to higher values, and the absolute amount decreases. This is in agreement with the visual findings: cluster diameters persist, cluster densities decrease, and more large distances appear inside the clusters. The action of trastuzumab is antagonistic to the action of NRG-1. The amount of distances characteristic for the unstimulated cell increases. The peak also shifts towards higher values, even if not as pronounced as after NRG-1 application. The shift may be a result of the trastuzumab itself. In contrast to the very small (some amino acids long) NRG-1 peptide, trastuzumab is a 150 kDa antibody [105]. The introduction of such a large molecule into a former densely-packed ErbB cluster will weaken the receptor package density. Again, the sketch at the bottom shows how the cluster protein depletion after NRG-1 stimulation and the cluster weakening after trastuzumab application alter the receptor locations subject to analysis. Finally, region 3 shows the receptor distances at values much larger than those arising in dense packed cluster accumulations. This part of the graph reflects receptors lying not in close vicinity. It becomes apparent that the total amount of receptor protein is limited. An increase of peak height at region 2 is compensated by an decreased trace level in region 3, and vice versa. Finally, the schematic situation shows the similarities inside the receptor populations. Even if the trace level differs between the unstimulated, the stimulated, and the attenuated case, there are no significant distance alterations visible in the observation window. It must be stated that the distance range between 150 and 250 nm is far out of reach for receptor interactions. Thus, the distance values from this range gain a certain randomness without arguable physiological relevance.

### 2.3. Trafficking Processes of ErbB Receptors after NRG-1 Stimulation, Trastuzumab Attenuation, and Irradiation

**Figure 2:**
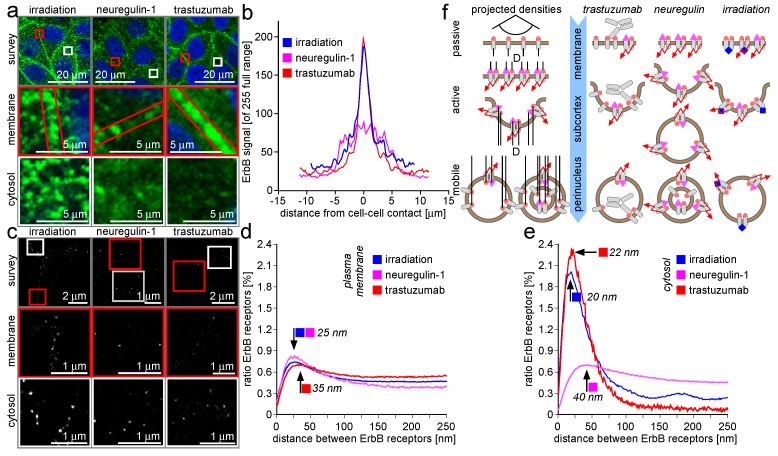
After the application of NRG-1, trastuzumab, or DNA-injuring irradiation, ErbB-2/3 receptors are mobilized from their initial residues in the plasma membrane and subject to retrograde trafficking. In this context, aside from a displacement in the μm range, alterations in the packing density on the nm level take place. (**a**) A nearly confluent monolayer of MCF-7 breast epithelial cells recorded by CLSM. DNA stain and antibody stain as well as color-coding as in Figure 1. The top row shows a cell survey with close up as shown in the insets (red and white boxes). The middle row shows cell-to-cell contact regions, while the bottom row shows the cytosol distant from the contacts. The columns show representative cells after irradiation (left), NRG-1 stimulation (middle), and trastuzumab attenuation (right). The red boxes in the middle column indicate the cell-to-cell contact region. For closer interpretation, see text. (**b**) Intensity profiles as described in Figure 1, but with the unstimulated case replaced by the irradiation case. (**c**) LM localization matrix visualizations with the selection according to the CLSM-based collection in (**a**). Again, surveys and details (red and white boxes) of plasma membrane and cytosol regions of the three cases of cell manipulation are given. (**d**,**e**) Inter-receptor distance plots for NRG-1 (purple), trastuzumab (red), and irradiation (blue) application—(**d**) for receptor clusters at the cell-to-cell contact region and (**e**) for the situation inside the cytosol. The black arrows indicate the local maxima of the curves. The color boxes indicate the manipulation resulting in the graph. (**f**) A collection of schemes clarifying the modes of ErbB-2/3 receptor dimers packing and retrograde trafficking. The optical effects arising when a plane plasma membrane-residing receptor population is undergoing budding, vesicle formation, and subsequent vesicle recombination. The linear arrangement of the receptor dimers is a side view onto the plasma membrane (top row). The straight membrane becomes curved during the process of budding (lobed structures immediately below). Four types of receptor loaded vesicles are displayed as circles at the bottom of collection (**f**). To the right of the blue arrow, a combination of different trafficking modalities as suggested by the CLSM and LM data are given. The arrow indicates the main traffic direction from the plasma membrane into the cytosol in the three cases of cell manipulation. For closer explanations, see text.

In Figure 2, the ErbB-2/3 receptor clustering is described with emphasis on the differences between cluster dynamics in the plasma membrane and packing dynamics inside vesicle membranes in the cytosol. For better understanding, one must regard the spatial compression effect when a three-dimensional membrane bud or vesicle is projected onto a two-dimensional image sensor plane (Figure 2f, left side). The projection results in an increase of the particle density by optical means, as compared to the optical projection of a plane plasma membrane area to the image—a process free of this imaging artifact. This does not mean that any increase in receptor density can be reduced to a pure optical projection artifact, but it must be regarded that the receptor density alterations during budding and vesicle formation result from a superposition of recording optics and cell physiology. This is prominently the case for multi-shell vesicles reported for receptor turnover [10].

As for Figure 1, the receptor cluster dynamics in the μm range as accessible by CLSM and in the nm range as analyzed by LM differ in a systematic fashion for the three cases of cell manipulation.

The CLSM survey images (Figure 2a, top row) recapitulate the finding from Figure 1 insofar as trastuzumab consolidates the ErbB receptor accumulation at the cell-to-cell contact regions while NRG-1 acts in an opposite fashion. The irradiation effect on ErbB receptors occupies an intermediate position. The cell border region does not seem to be depleted from ErbB receptors as in the NRG-1 case, but the border accumulation is less pronounced as compared to the trastuzumab case. A closer look confirms this finding. The cell border ErbB receptor population is much more dense after trastuzumab application and more depleted of ErbB protein after NRG-1 application than in the irradiation case. Most interesting is that these relations are not conserved in the cytosol. While the cytosol of trastuzumab-treated MCF-7 cells contains a low ErbB protein amount and the cytosolic protein amount after NRG-1 application is also increased, the cytosolic ErbB protein amount after irradiation should rather recapitulate the trastuzumab situation. The ErbB receptors residing on the cell borders should decrease the amount of cytosolic protein. In fact, the irradiation results in a protein concentration similar to that after NRG-1 application.

The intensity line plots in Figure 2b confirm the intermediate effect of irradiation on the ErbB receptor mobilization. The irradiation effect recapitulates the trastuzumab effect at the cell border, but ranges between the NRG-1 and trastuzumab in the cytosol.

The most impressive result of the LM examinations is the very pronounced effect of trastuzumab and irradiation on receptor packing density in the cytosol, while at the same time recapitulating the effects of the three manipulations at the cell border as already indicated in Figure 1. In Figure 2c, LM matrix visualizations of typical cell regions in survey, for the cell border area and for the cytosol are given after cell manipulation by irradiation, NRG-1, and trastuzumab application, respectively. In the following, a comparison of ErbB-2/3 clusters in the plasma membrane and ErbB-2/3 packing in the cytosol is compared, assuming that signals refer to dimers.

LM data do not recapitulate CLSM findings in a one-to-one fashion. In CLSM, the accumulation of vesicles in the cytosol after NRG-1 application is accompanied by a depletion of the ErbB-2/3 cluster population at the cell border. In contrast, accumulating ErbB-2-containing vesicles in the cytosol after irradiation does not remarkably reduce the amount of ErbB-2/3 clusters at the cell border. This discrepancy is recapitulated on the nm scale by LM data. After stimulation with NRG-1, the ErbB-2/3 cluster distances are shifted to higher values, indicating a decrease of the cluster density and concomitant loss of Erb-2/3 dimers by bud formation and entering the cytosol in vesicles. A closer look at the resulting cytosolic fraction reveals a broad receptor density distribution with only a small peak at small distances and a long plateau at medium and large distances. This means that the mobilized ErbB-2/3 receptor dimers are packed in an inhomogeneous manner, presumably into vesicles with a receptor packing that is not carefully regulated [11].

The same examination done for the ErbB-2/3 receptor dynamics after irradiation reveals another situation. As given by the CLSM image, the receptor cluster population density shifts slightly towards higher values, indicating a depletion of the plasma membrane-located clusters from receptors but without losing the clusters themselves. The cluster density decreases in spite of a cluster loss in total. Additionally, the cytosolic vesicular ErbB-2/3 containers are different in the irradiation case as compared to the NRG-1 case. Inside the cytosolic vesicle fraction, ErbB-2/3 receptors are accumulated at high packing densities (Figure 2e), while there are nearly no cases with receptors in a loose packing (i.e., the cytosol-derived density plot trace shows no plateau at medium and high distances).

The authors interpret this particular individuality of receptor density distribution at the cell border and in the cytosol in that there exists a systematic difference between the budding and vesicle formation processes between irradiation and NRG-1 application, respectively. As an additional finding, there is a slight peak in the ErbB-2 accumulation after NRG-1 application in the cytosol at a distance of around 175 nm. This may indicate an additional organization level, leading to both low and high distance ErbB-2 receptor accumulations.

The LM analysis after trastuzumab application recapitulates the CLSM finding in that thus-modified ErbB-2 receptors accumulate at the cell border. The increased absolute amount of protein induces a somewhat broader density distribution—an effect of antibody sterics: The therapeutic trastuzumab antibody molecules as well as the antibody molecules used for staining co-localize with the ErbB receptors and require additional space inside the accumulates. This increases the distances between some but not all ErbB receptor molecules and as a result additional distances appear. The cytosolic fraction is also of outstanding interest for trastuzumab application. The CLSM shows only a few vesicles in the cytosol, but the LM data (Figure 2e) show that the containing ErbB-2/3 receptors are packed in a high density. No loosely-packed vesicles occur as indicated by the trastuzumab trace in Figure 2e.

In Figure 2f (right side), the individual container properties of the cytosolic vesicle after trastuzumab-, NRG-1-, and irradiation-based manipulation are visualized for the plasma membrane, the membrane-neighboring (i.e., the subcortical) cytosol fraction and the cytosol deeper inside the cell (i.e., perinuclear or around the cell nucleus). The rare vesicles contain ErbB-2 receptors in a packing much higher than in the NRG-1 case, but somewhat decreased as compared to the irradiation case. This is due to the antibody content inside the vesicle lumen. For NRG-1 application, an inhomogeneous vesicle population with varying receptor packing density exists. Presumably, multiple layered vesicles with more than one lipid bilayer as shown in transmission electron microscopy are responsible for this finding [10]. In the irradiation case, the receptors are also packed in high density. No antibody inside the vesicular lumen disturbs the packing, and in this way, the density reaches slightly higher values as compared to the trastuzumab situation.

In conclusion, the LM-based analysis of the cytosolic ErbB-2/3 receptor dimer fraction gives rise to the assumption that the cell reaction to NRG-1, irradiation, and trastuzumab application induces individual regulation processes which result in different vesicle populations.

### 2.4. ErbB-2/3 Receptor Dimers and Gap Junctions Share Common Mobilization Characteristics

**Figure 3:**
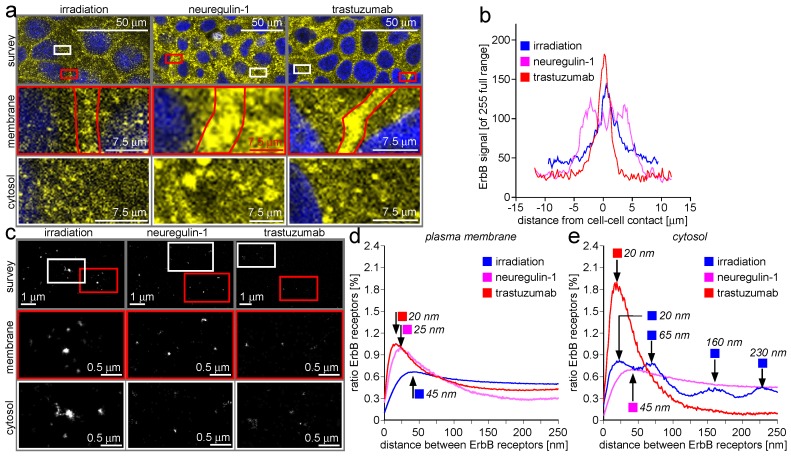
The manipulation of ErbB receptors with NRG-1, trastuzumab, or DNA-injuring irradiation co-mobilizes gap junction connexons from their initial residues in the plasma membrane and induces retrograde trafficking. The displacement in the μm and nm range shows characteristics exclusive for gap junction mobilization, even if ErbB receptors are the target of stimulation. (**a**) A nearly confluent monolayer of MCF-7 breast epithelial cells recorded by CLSM. The blue nuclear DNA stain as above. The antibody stain (yellow) against connexin-43 according to the same protocol as for ErbB receptors. The top row shows a cell survey with close up shown in the insets (red and white boxes). The middle row shows cell-to-cell contact regions, while the bottom row shows the cytosol distant from the contacts. The columns shows representative cells after irradiation (left), NRG-1 stimulation (middle), and trastuzumab attenuation (right). The red boxes in the middle column indicate the cell-to-cell contact region. (**b**) Intensity profiles as described in Figure 1, but with the unstimulated case replaced by the irradiation case. (**c**) LM localization matrix visualizations with the selection according to the CLSM-based collection in (**a**). Again, surveys and details (red and white boxes) of plasma membrane and cytosol regions of the three cases of cell manipulation are given. (**d**,**e**) Connexon distance plots for NRG-1 (purple), trastuzumab (red), and irradiation (blue) application—(**d**) for receptor clusters at the cell-to-cell contact region and (**e**) for the situation inside the cytosol. The black arrows indicate the local maxima of the curves. The color boxes indicate the manipulation resulting in the graph.

Connexons do not have any binding moieties for NRG-1 or trastuzumab, and also do not show any irradiation sensitivity. In conclusion, the reaction of ErbB-2/3 receptor-mediating compounds must be due to an indirect effect (see above for explanation). In this context, it must be stated that the stimulation has an indirect characteristic. The type of agonist and antagonist focus on the manipulation of ErbB-2/3 receptors. Application protocols for agonist and antagonist—like dose and application time—also do not address the requirements for connexin-43. In all reported experiments, the combination of connexin-43 packing density together with the transport into the cytosol are not examined, and this stimulated us to take a closer look at this aspect. In Figure 3, the dynamics of connexin-43 protein after application of NRG-1, trastuzumab, and irradiation is given.

In the CLSM recordings, the mobilization of connexon recapitulates ErbB receptor mobilization at first glance (Figure 3a). A consolidation of connexon accumulation at the cell border is stimulated by trastuzumab application, while irradiation depletes the cell border of connexons. In contrast to the border-located ErbB receptor behavior after NRG-1 application, connexons reside at the cell border. Additionally, for the cytosol, the CLSM results are not as conclusive for connexons as for ErbB receptors. In all three cases, the cytosol appears to be comparably loaded with vesicles. The vesicle population after irradiation appears to be more coagulated compared to the two other cases, but this might be a local effect of only minor general significance. In Figure 3b, the intensity plots show clear differences for the three manipulations. Under the influence of trastuzumab, connexons form a well-consolidated accumulation that is sharply restricted to the cell border. The distribution of connexons after irradiating the cells results in a loss of the steep distribution and a concomitant decrease of the peak maximum, because the connexons are mobilized away from the cell border. This effect is even more pronounced after NRG-1 application.

The LM localization matrix visual reconstructions in the form of images contain less interpretable information than the connexin-43 protein distance plots given for the plasma membrane (Figure 3d) and the cytosol (Figure 3e). All traces show connexin-43 distances after manipulating ErbB receptors with trastuzumab, NRG-1, or by DNA-injuring irradiation. In the plasma membrane population, irradiation induces a peak shift towards higher values as compared to the other two manipulation modes. This is remarkable, because on the basis of molecular biological information, NRG-1 and trastuzumab act on ErbB receptors antagonistically. In the present case, NRG-1 does not induce a peak shift and does not induce a plateau-like distance distribution at high values as shown for ErbB receptors (see data above). In the cytosolic fraction given in Figure 3e, the trastuzumab vesicles shown in the CLSM recordings contain densely packed connexin-43 proteins (red trace). The packing density analysis is limited by the lower resolution value induced by the size of the antibodies used in indirect antibody staining (see above for closer explanation). Accumulations at higher distance values are lacking, as expressed by the steep decrease of the distance distribution plot. In contrast, the cytosolic connexin-43 distance distribution after NRG-1 application recapitulates the ErbB receptor situation (purple trace). This means that the antagonistic action of trastuzumab to NRG-1 on ErbB-2/3 receptors is recapitulated by connexin-43 protein molecules (and presumably connexons) in the cytosol, but not at the cell border. Of utmost interest is the connexin-43 distance distribution in the cytosol after irradiation (Figure 3e, blue trace), which shows a series of peaks at low (20 nm), medium (65 nm), and high (160 and 230 nm) distances. The 65, 160, and 230 nm distances are far above the connexon size. It can be assumed that the responsible structures are not gap junction-based but derive from vesicle size. This implies that the sizes of the vesicles containing connexin-43 after irradiation are random, but fall into different size populations with each population discriminated by the others. An alternative explanation might again be the occurrence of multiple layered vesicles as described above.

## 3. Discussion

The presented work examines the dynamics of receptor tyrosine kinases of the ErbB family upon stimulation with the canonical agonist neuregulin-1 (NRG-1) and after receptor attenuation by the therapeutic antibody trastuzumab. Additionally, receptor dynamics after DNA-injuring irradiation is recorded. Besides the ErbB-2 receptors as the primary target of examination, the concomitant dynamics of gap junctions after manipulation of ErbB receptors have been recorded. The cell line used for investigations (MCF-7) expresses both ErbB-2 and ErbB-3 receptors, as well as connexin-43-containing gap junctions. After the application of irradiation, NRG-1, or trastuzumab to the otherwise-untouched cells, fixation at life stabilization conditions conserved the cell reaction state. This state was subsequently isolated and identified by indirect immuno-stain and fluorescence microscopy. Consequently, the receptor dynamics described are result of a superposition of life cell stimulation, epitope staining, and CLSM as well as LM microscopy imaging.

### 3.1. ErbB Receptor and Connexin-43 Protein Visualization and Measurement by CLSM and LM

The modes of increasing the optical resolution during CLSM imaging and circumventing the optical resolution limit by LM matrix recording resulted in particular and individual information about the specimen. Both methods have in common that a conventional image formation by optical interference is replaced by methods with the capacity to acquire fine spatial details of the specimen. CLSM reduces the diffraction of the image recording light spot by cutting off the intermediate image. The accompanying loss of image information is compensated for by scanning the spot across the specimen and recording the image as a line- and frame-wise reconstruction. For LM, an image series is analyzed for spatially- and temporally-isolated fluorescence emission events, and the image is put together from the isolated points as intermittently deposited in a mathematical matrix (see Table S1).

In the two resulting specimen records, the main difference can be assigned as continuous versus discontinuous specimen information. Both representations illustrate certain properties of the ErbB receptor and the connexin-43 constitution and dynamics. While the CLSM displays the continuous aspect of receptor accumulations in plasma membrane and cytosol, it refers to the functional manifestation of ErbB RTKs and gap junctions. Kinase activity and gap junction communication are effective on a μm range. Material entering the cytosol across gap junctions and phosphorylated proteins is prone to diffusion, and in this way the local action of the membrane channels and RTKs is continuously displaced into the environment.

In contrast, the results of LM specimen recording identify the set of spatial positions of ErbB receptors and gap junctions as individual manifestations. Each identified protein is displayed as an individual point in a mathematical matrix or in a reconstructed image situation. This approach emphasizes the character of ErbB-2 and connexin-43 as individual protein molecules. The ability to look at the protein distribution in the nm range facilitates the examination of individual inter-protein distances. This information can be correlated with molecular biological stimulation and long-range movement.

It can be stated that protein local accumulation, protein long (μm) range displacement by trafficking, and alterations in protein short (nm) range organization interact on a cell physiological basis.

### 3.2. ErbB Receptor Membrane Clustering Modified by NRG-1 or Trastuzumab as Compared to the Unstimulated Situation

After the application of the dimerization agonist NRG-1 or the dimerization antagonist trastuzumab, the local accumulation and the trafficking and clustering of ErbB-2/3 dimers is altered. Accumulation and trafficking can be recorded by CLSM, while cluster dynamics can be assessed by LM. Both microscopy examinations follow the fluorescence distribution after a specific antibody-to-epitope complex formation. Again, this process is of minor spatial influence in the case of CLSM, but exaggerates a pronounced effect interacting with the cluster dynamics as recorded by LM.

ErbB receptor plasma membrane accumulation or dissipation and related receptor trafficking in the cytosol after neuregulin-1 or trastuzumab application reflect the cell biological activity of the ErbB-2/3 receptors in transducing the neuregulin-1 stimulation or the suppression of this activity by trastuzumab. Dimerized (i.e., activated) ErbB RTKs participate in a set of phosphorylation-based signal cascades. Upon stimulation, cells differentiate, alternate energy metabolism, modulate the levels of stress factors (such as oxidation compounds), and become prone to epigenetic pattern alterations. Activated cascades immediately cover the MAPK, the AKT, and the PKC*ϵ* regulation cascades. Objects of indirect regulation are STATs, CREB and components of the regulators for proliferation and programmed cell death (for explanation of the abbreviations used here refer to the table at the end of this document). Cell migration might also be concerned.

The activation or attenuation of ErbB receptors is accompanied by population mobilization, but also by alterations in characteristic population density (or distance) distributions. Receptor density frequently correlates with receptor response amplitude. Increased packing densities of membrane receptors result in a higher signal-to-noise ratio and an accelerated receptor reaction. While this statement meets reality, for example, in the case of electrical membrane channels, the situation after RTK stimulation is more complicated. In the present case, it must be regarded that ErbB-2/3 dimers not only act inside the plasma membrane but are also shuttled to the nucleus to participate in DNA repair regulation. This means that an increase in receptor density might have an ambivalent effect, depending on cell locus and agonist versus antagonist stimulation. Trastuzumab decreases ErbB phosphorylation activity, but increases the plasma membrane fraction density by prohibiting receptor nucleograde trafficking. The activation by NRG-1 and subsequent vesicle-based receptor trafficking induces a depletion of the plasma membrane fraction with an accompanying increase in receptor distances. Further, receptor trafficking across the cytosol results in a broad distance distribution. This reflects the packing of RTKs in transport vesicles.

For receptor tyrosine kinases (RTKs) of the Epidermal Growth Factor Receptor (EGFR)/ErbB-*n* family (with *n* = 2 − 4), activation of the kinase function coincides with receptor accumulation. This accumulation covers the basic receptor dimer aggregation with ErbB 2/3, 2/4, and 3/4 heterodimers, and 3/3 and 4/4 homodimers known as carriers for kinase activity [14]. In general, it can be stated that in the case of membrane receptor activation, aside from the above-mentioned dimerization, the accumulation receptor dimers in groups can appear [11]. Such aggregates are known for several ion channels, like sodium and potassium channels, but also calcium channels of the dihydro-pyridine type in the plasma membrane and for the ryanodine-type calcium channels in the endoplasmic or sarcoplasmic reticulum membrane [107]. Additionally, the T-cell receptor and auxiliary proteins in the immunological synapse are clustered in the cell membrane [97]. In front of this background the question appears in how far activated ErbB receptor pairs of the 2/3 type aggregate in clusters, and further if the clusters cover a structural order organization principle different from the basic receptor dimerization. Clusters of increased size, high packing density, and a well-organized receptor population are capable of faster stimulus reaction cycles and increased signal amplitudes by an unchanged background activity level [33]. For comparison, the organization state of ryanodine receptors in the sarcolemma of very fast reacting muscle cells or cardiac myocytes approximates the regularity of two-dimensional protein crystals, while the same channel type in slow reacting cells is randomly distributed [108].

For LM recording, the distance between the emission burst center of the fluorescent dye molecule and the ErbB-2 epitope is a critical prerequisite for close localization. The more distant the dye molecule is, the larger is the uncertainty for the epitope localization. Due to the production process, the secondary antibody as purified from the blood serum of a donor organism directs against a series of epitopes on the primary antibody. Each epitope is located in a non-predictable region on the primary antibody. Further, the dye loading by coupling to serine or glutamate side chain residues anywhere on the secondary antibody further increases the uncertainty for the dye-to-ErbB-2 epitope distance. This situation is sketched in Figure 1c (top row, left). This value must be doubled, because an antibody set will rotate and describe a circle of uncertainty with an ErbB-2 epitope in the center. In summary, the inherent spatial variability of the indirect immune staining introduces a localization uncertainty by 20–25 nm. This is a principle limitation for localization.

The spatial independence of ErbB-2 epitope location and fluorescence burst location can also introduce both false positive and false negative indicators for receptor clustering. Refer to Figure 1c (bottom row) for this relationship. An inactive isolated ErbB-2 monomer will appear as isolated and not clustered in the LM matrix, criteria for designating it as inactive by spatial isolation. In contrast, a (small) receptor cluster might contain active dimers, but the antibody stain steric situation does not account for a complete staining of the densely-packed ErbB-2 receptors inside the cluster. In this case, the ratio of active dimers is underestimated. In an opposite case, the close spatial approximation of two secondary antibodies both binding two distant not-clustered ErbB-2 epitopes will result in a false positive (i.e., clustered) ErbB-2 receptor population. This problem can be addressed by the double staining of ErbB-2 and ErbB-3 receptors with LM recording and subsequent proximity analysis, but such an examination is vulnerable against mechanical drift during image acquisition and cannot discriminate between isolated receptors and receptor dimers not stained for both epitopes due to steric hindrance or limited antibody affinities, for example.

### 3.3. Trafficking Processes of ErbB Receptors after NRG-1 Stimulation, Trastuzumab Attenuation, and Irradiation

ErbB receptors are trans-membrane proteins. This means that they can be affected by extracellular signals like NRG-1 or trastuzumab, as well as intracellular signals like tagging, phosphorylation, or complex formation with protein binding proteins like HSP-90, HSP-70, or importin-1β. Additionally, the binding of an extracellular agonist might increase the tendency for binding by an intracellular protein.

The CLSM imaging shows alterations in ErbB protein distribution between the plasma membrane and the cytosol after the application of NRG-1, trastuzumab, or irradiation. In brief, while trastuzumab induces a consolidation of the ErbB membrane fraction, NRG-1 and irradiation stimulate the release of ErbB protein from the plasma membrane into the cytosol. Even more interesting than the mobilization in principal are alterations in receptor density together with receptor trafficking. On the basis of local (membrane vs. cytosol) packing density, a split of the receptor population into different packing classes on the basis of position inside the cell can be seen. The local density dynamics reflect the mode of receptor movement. In the plasma membrane, receptors accumulate in plaques. After plaque formation, the process of membrane bud formation starts the cytosolic receptor retrograde trafficking procedure. The buds form vesicles containing the receptors in characteristic densities and transport them across the cytosol. This procedure can be seen in the pronounced difference when the plasma membrane receptor distance distribution faces the cytosolic one. While at the plasma membrane a broad spectrum of receptor densities for all three cases of manipulation (NRG-1, trastuzumab, irradiation) indicates a minor reaction, the receptor densities in the cytosol are an expression of extended receptor packing activity. Comparing the cytosolic packing density distributions after NRG-1 application with irradiation reveals two individual packing and trafficking modes. While NRG-1 packing covers a broad density distribution, irradiation-induced packing results in a sharp density peak similar to the cytosol receptor residual population in the case of trastuzumab application.

This finding gives rise to the assumption that ErbB receptors are subdued to individual packing processes dependent on the mode of receptor activation.

The ErbB receptor family members are receptor tyrosine kinases (RTKs). This means that an extracellular stimulus induces an intracellular phosphorylation activity [63]. Thus, ErbB receptors are trans-membrane proteins with an outer agonist (or receptor subtype)-binding domain and an inner kinase domain or a functional component of such a domain [109]. In the latter case, dimerization of two receptors starts kinase activity [110]. This consideration does not include that the place of agonist action and kinase reaction are the same. In the case of ErbB-2/3, receptor dimers for at least one intracellular locus of kinase activity is not near the plasma membrane (i.e., the cellular sub-cortical cytosol), but inside the nucleus or in the cytosol in the immediate vicinity [12,76]. Consequently, activated ErbB-2/3 receptors must be subject to retrograde or nucleograde trafficking, a non-random transport process from subcortical cytosol to the cytosol around the cell nucleus [17,111]. ErbB-2/3 dimer trafficking will be function conserving only as long as the dimers keep their native tertiary and quaternary protein structure. For membrane proteins, this implicates the need to conserve the surrounding lipid bi-layer and presumably a regulative and spatially-stabilizing membrane raft [112]. By geometrical means, it may concluded that ErbB receptor retrograde trafficking will be dependent on plasma membrane budding and cytosolic vesicle formation [37,58]. The required deterministic character of nucleograde trafficking requires the participation of cytoskeleton elements and networks and proteins mediating the mechanical contact between the vesicle and its transporter [85,113,114]. The findings and processes given in Figure 2 illustrate this situation for MCF-7 breast epithelial cells after stimulation by NRG-1 or attenuation by trastuzumab, as well as after DNA damage-inducing irradiation.

The appearance of the intracellular membrane apparatus seems to be chaotic with individual vesicles in high density packed in the cytosol, but the process is a highly regulated procedure with protein tags on the vesicle surface for vesicle state and fate [61]. Besides the geometrical pathway a vesicle follows across the cell, the functional environment changes and the vesicle content and size is also altered by fusion processes with other vesicles and by digestion [113].

In case of ErbB nucleograde trafficking, a terminal point is the fusion of the vesicle with the nuclear envelope and the transfer of receptors across the nuclear pore complexes (NPCs) into the nuclear matrix [115]. The ErbB-2/3 dimers are discussed to participate in phosphorylation-based regulation of protein activity inside the nucleus [116]. Some of the regulation activities are involved in DNA repair processes after irradiation-induced DNA lesions [117]. Another stimulation for retrograde trafficking may be the receptor turnover and the removal of receptors according to their half-life [54]. This should result in a trafficking background level with a superposition of event-induced trafficking on top.

### 3.4. ErbB-2/3 Receptor Dimers and Gap Junctions Share Common Mobilization Characteristics

Parallels in the mobilization dynamics of ErbB-2/3 receptor dimers and gap junctions shed light on the underlying cytosolic kinase cascades. They also reflect that not only ErbB RTKs, but also gap junctions are key players in the progression and metastatic activity of breast cancer development. In contrast to breast epithelium, gap junctions in the nervous system or in the cardiovascular system have a substantial impact on tissue integrity and function. Such tissues are also subject to ErbB receptor NRG-1-based regulation. The finding of concomitant ErbB and gap junction mobilization by NRG-1-, trastuzumab-, and irradiation-based stimulation poses the question as to whether the systemic release of NRG-1 affects spatial and functional distant cells and tissues in a paracrine fashion. The question of whether accidental irradiation of, for example, heart tissue during anti-cancer therapy induces heart failures must be extended in that the chemotherapeutic manipulation (by trastuzumab) of ErbB receptors in malignant tissue is systematically accompanied by affecting the gap junction distribution in distant non-malignant tissues like the heart. Alterations in cardiac fatty acid turn over and storage, the mitochondria-based energy metabolism, and the signal conducting pathways perturbations are side-effects during anthracyclin/irradiation/therapeutic antibody combination therapy.

The dimerization of ErbB-2 with ErbB-3 monomers activates the tyrosine kinase reaction centers on the receptor cytosolic face. One substrate of this ErbB RTK is protein kinase B (PKB, also called AKT). This cascade induces the nucleograde transport of RTKs of the Epidermal Growth Factor Receptor (EGFR) type as well as ErbBs [118]. Another branch of kinase cascade covers the mobilization of gap junctions by a protein kinase C (PKC)-mediated signaling [83]. This overlapping induction of signal pathways induces a concomitant mobilization of gap junctions by the stimulation of ErbB receptors.

Gap junctions are accumulations of connexons inside regular fields in the plasma membrane [119]. Connexons are hexameric protein quaternary structures with a channel domain in the center of the hexamer symmetry long axis of the structure [120]. Connexons are made from several proteins, with connexins being the main component that are responsible for the overall appearance of connexons [121]. Connexin-43 is a widespread subtype and is used for gap junction staining in this examination. In MCF-7 breast epithelial cells, gap junctions participate in the communication between adjacent cells and with the environment [122,123]. They also participate in electrical signal reception during environmental stimulation. Gap junctions also play a role in the development of several cancer types, with breast cancer as a prominent one [124,125,126,127,128].

Despite their size, gap junctions are highly dynamic aggregates with short half-life and a high lateral mobility inside the plasma membrane [46,129,130]. This high lateral mobility is accompanied by a substantial anterograde shuttle mechanism to transport new connexin protein to the plasma membrane [131,132]. This anterograde transport is balanced by a retrograde trafficking which regulates the amount of protein by proteolysis [48,51,133,134]. Both retrograde and anterograde transport pathways are orthogonal with respect to the cortical (also called lateral) transport direction inside the plasma membrane.

Besides the kinase pathway interaction sketched above, the motivation also derives from the role of ErbB-2/4 and 4/4 receptor dimers in the development of the embryonic tissue and from the role of NRG-1 during the differentiation of embryonic stem cells into cardiac myocytes—a procedure accompanied by extensive alterations in the gap junction appearance [84,135,136,137,138,139]. Further, after therapeutic or non-intended irradiation and cancer therapy, the gap junction-related behavior of cells, tissues, and organs can be altered [140,141]. Finally, the application of trastuzumab during cancer therapy can induce a mobilization of gap junctions along the cell surface, and in this way modify the signal perception pattern. Additionally, physiological processes on different time scales (minutes to weeks) are affected [142,143,144,145]. Such effects can be disabled by NRG-1 application [64,146,147,148].

In several cases, the malignancy of breast cells coincides with a mismatch in the expression and handling of receptors of the ErbB family. This phenomenon is not only synchronized with mismatches in gap junction physiology, but both protein populations turnover and function are closely coupled [149,150]. This correlation is a possible reason for the influence of chemotherapeutics which focus on ErbB receptors as their main target but also manipulate gap junctions in conventional breast tumor cells and in tumor stem cells [151,152,153]. In particular, it could be shown that the apoptosis activity level of conjugated linoleic acid via stimulation of caspase-3 in MCF-7 breast cancer cells is dependent on gap junction permeability [154]. Modulators of gap junction permeability derive from pesticides and polychlorinated aromatic compounds, but hormones like estradiol can also induce carcinogenesis in breast epithelial cells with increased ErbB receptor number [155,156]. In addition, the gap junction functionality not only covers the correct stimulation via soluble compounds, but is also involved in contact-based cell-to-cell communication of breast epithelial cells, cancer stem cells, and endothelial cells [157,158]. Consequently, markers for gap junction-based cell communication are investigated for their function as targets in anticancer therapy [159].

## 4. Materials and Methods

### 4.1. Cell Culture and Cell Manipulation

#### 4.1.1. Cell Culture

Michigan Cancer Foundation cell line 7 (MCF-7) cells were cultivated in Roswell Park Memorial Institute Medium number 1640 (RPMI-1640) with 2 mM L-Glutamine, 100 Units/mL Penicillin, 100 μg/mL Streptomycin (all Thermofisher, Waltham, MA, USA), 10% fetal calf serum (FCS, Biochrom, Berlin, Germany), and 25 mM 2-(4-(2-Hydroxyethyl)-1-piperazinyl)-ethansulfonic acid (HEPES) (Carl Roth, Karlsruhe, Germany). The cells were cultivated on 19 mm × 19 mm lime glass cover slides, thickness class 1 (Wenzel Gläser, Berlin, Germany), in Cellstar six-well plates (Greiner Bio-One International, Frickenhausen, Germany) up to 75% monolayer confluence before microscopy specimen preparation. Environmental conditions were H${}_{2}$O-saturated atmosphere at 37 ${}^{\circ }$C and 5% CO${}_{2}$.

#### 4.1.2. Stimulation of MCF-7 Cells with Neuregulin-1

MCF-7 cells were cultivated at 50% confluence prior to NRG-1 application for 12 h in complete RPMI 1640 medium with FCS content reduced to 0.5%. Replace by complete RPMI 1640 medium without FCS for additional 3 h. Replace by complete RPMI 1640 medium without FCS but with 330 nM NRG-1 (Abcam, Berlin, Germany) for 1 h. Replace by complete medium RPMI 1640 with 10% FCS without NRG-1 for one hour before cell fixation.

#### 4.1.3. Stimulation of MCF-7 Cells with Trastuzumab

Replace FCS concentration as described above. Replace by complete RPMI 1640 medium without FCS but with 172 nM trastuzumab (Roche Diagnostics, Munich, Germany) for 1 h. Replace by complete medium RPMI 1640 with 10% FCS without NRG-1 for one hour before cell fixation.

#### 4.1.4. Irradiation of MCF-7 Cells

Cell monolayers at 75% confluence on cover slides in six-well plates (see above for details) were irradiated with 4 Gy total dose by 6 MeV photons using an Artiste linear accelerator (Siemens, Munich, Germany). Cells were transported at 37 ${}^{\circ }$C and irradiated at ambient temperature (approx. 20–25 ${}^{\circ }$C). Cells were kept at cell culture ambient conditions and fixed at 45–60 min after irradiation (see Figure S2).

### 4.2. Microscopy Specimen Preparation

If not otherwise stated, all chemicals were from Thermofisher, Waltham, USA, and phosphate buffered saline (PBS) was used as solvent. For further preparation, the cells were briefly rinsed with PBS supplemented with 1 μM calcium and 2 μM magnesium at 37 ${}^{\circ }$C and fixed in 4% formaldehyde for one hour. All antibodies were diluted in PBS without calcium and magnesium supplemented with 1% (*w/v*) bovine serum albumin fraction V, (ethanol precipitated, fatty acid free) (No. 8806, Sigma Aldrich, Munich, Germany) and 0.1% sodium azide (Merck GmbH, Darmstadt, Germany). Phalloidine labelled with Alexa-647 (1 μg/mL) and DAPI (50 ng/mL) were dissolved in demineralized water with 0.1% sodium azide. All washing steps were performed in PBS. Specimens were kept for storage in PBS with 0.1% sodium azide. The following antibodies were applied in a 1:100-fold dilution: mouse monoclonal anti-c-ErbB-2 primary antibody (No. E2777, Sigma Aldrich, Munich, Germany), rabbit polyclonal anti-Connexin-43/GJA1 primary antibody (No. ab11370, Cambridge, United Kingdom), goat polyclonal anti-mouse secondary antibody conjugated to Alexa-555 (No. 150118, ibid), donkey polyclonal anti-rabbit secondary antibody conjugated to Alexa-488 (No. 150061, ibid). Consecutive order of application: primary antibodies anti-c-ErbB-2 (AB-I) followed by secondary antibodies anti-AB-I. Intermediate fixation was performed in 2% formaldehyde solution at room temperature for 1 h. Primary antibodies anti-Connexin-43/GJA1 (AB-II) followed by secondary antibodies anti-AB-II. Phalloidine application was followed by DAPI application. Every application step took 12 h at 4 ${}^{\circ }$C followed by a five-times wash for 5 min at ambient temperature. The specimens were embedded in Prolong Gold embedding medium (Thermofisher, Waltham, MA, USA).

### 4.3. Microscopy Data Recording

#### 4.3.1. Confocal Laser Scanning Microscopy (CLSM)

The Leica TCS SP5 DMI6000 inverted microscope was used with an HCX PL APO lambda blue 63 × 1.4 Oil UV-type objective lens. Four fluorescence acquisition windows (I.–IV.) and one bright field transmission window (V.) were defined as below: I. Excitation 405 nm, acquisition 414–483 nm for nuclear DAPI fluorescence; II. Excitation 488 nm, acquisition 501–551 nm for connexin-43/JGA1 Alexa-488 fluorescence; III. Excitation 561 nm, acquisition 570–623 nm for c-ErbB-2 Alexa-555 fluorescence; IV. Excitation 633 nm, acquisition 642–732 nm for f-actin phalloidin Alexa 647 fluorescence; V. Excitation with accumulated excitation, specimen absorbance acquisition. Frame size 246 μm × 246 μm with 2048 pixels × 2048 pixels discrimination and the pinhole diameter fixed at one airy unit (equals 95.5 μm aperture sizes). Simultaneous channel acquisition in one-directional scan mode with eightfold line averaging resulting in a total pixel dwell time of 80 μs. Signal calibration was done by photomultiplier tube (PMT) detector excitation using the manufacturer’s calibration look-up table (Leica green->glow-red->blue LUT). Each channel recording was exported as 8-bit grey-scale uncompressed image in TIFF format. Color overlays were done in Image J.

#### 4.3.2. Localization Microscopy (LM)

In the epi-illumination fluorescence microscope setup, a LightHub-6 (Omincron Laserage, Rodgau-Dudenhofen, Germany) boxed laser combining module put the lasers LuxX 405 nm / 60 mW, 488 nm / 200 mW, and 647nm / 140 mW (emission wavelength / light emission power at laser output) onto a common aperture. A beam expanding telescope (Edmund Optics, Karlsruhe, Germany) fed the beam across a Gaussian to a top hat shaping element (Eksma Optics, Vilnius, Lithuania) into the rear aperture of a 100 × 1.4 oil immersion objective lens (Zeiss, Jena, Germany). The epi-illumination path contained three alternative filter sets to discriminate excitation and emission wavelengths with values (emission main | half maximum edge | emission main/width, values in nm): 390/40 | 420 | 452/45 and 482/18 | 488 | 525/50 and 642/20 | 659 | 705/72 (AHF Analysentechnik, Tübingen-Pfrondorf, Germany). The image series (1500 images typical, frame rate 30 Hz, full average mode) were collected by an emCCD camera (Andor via LOT-QuantumDesign, Darmstadt, Germany) and saved to a PC in 16-bit grey-scale mode in TIFF image stack format. Pixel size was 102 μm, resulting in a recorded specimen region of interest (ROI) of 37 μm × 37 μm.

### 4.4. Data Processing

The line plot intensity data from CLSM image recordings were collected using the standard line plot tool in the Image J software package (National Institute of Health, Bethesda, MD, USA). LM data processing and matrix operations were done in the Matlab software environment in script interpreter mode (Mathworks, Aachen, Germany). In-house designed scripts startSPDM version 4 and Autoclusters were used for matrix generation from image stacks and extraction of distance distribution spectra as histograms. Origin 2015 (Originlab, Northampton, MA, USA) was used for the generation of envelope function of the distance distribution histograms. The Matlab scripts formulating startSPDM version 4 and Autoclusters are available on request from Michael Hausmann (hausmann(AT)kip.uni-heidelberg.de).

## Supplementary Materials

Supplementary materials can be found at www.mdpi.com/1422-0067/18/2/362/s1.

## Abbreviations

The following abbreviations are used in this manuscript:

AA(s)	Amino Acid(s)
AKT	Gene locus (and protein) family of serine/threonine specific kinases
CLSM	Confocal laser scanning microscopy
CREB	cAMP response element-binding protein
DAPI	4′,6-diamidino-2-phenylindole
DNA-PKs	DNA-dependent protein kinase, catalytic subunit
FP(s)	Fluorescent Protein(s)
FT	Fourier transform
HEPES	*N*-2-Hydroxyethyl piperazine-*N*-2-ethane sulphonic acid
HSP(s)	Heat Shock Protein(s)
IgG	Immuno globulin (of the type) G
LM	Localization microscopy
MAPK	Mitogen Associated Protein Kinase
NPC(s)	Nuclear Pore Complexe(s)
NRG-1	Neuregulin-1
OTF	Optical (or: Objective lens) Transfer Function
PALM	Photo Activated Localization Microscopy
PKB	Protein Kinase B (Gene product of AKT gene locus)
PKC*ϵ*	Protein Kinase C epsilon
PI3K	Phosphoinositol Tris Phosphate Kinase
ROI(s)	Region(s) of interest
ROS	Reactive Oxygen Species
RTK(s)	Receptor Tyrosin Kinase(s)
STAT	Signal Transducer (and) Activator (of) Transcription
STORM	Stochastic Optical Reconstruction Microscopy
