# Supplementary material for: Localisation Microscopy of Breast Epithelial ErbB-2 Receptors and Gap Junctions: Trafficking after γ-Irradiation, Neuregulin-1β, and Trastuzumab Application
Source: Int J Mol Sci. 2017 Feb 9;18(2):362. doi: 10.3390/ijms18020362 (PMC5343897; doi:10.3390/ijms18020362)
Supplement: Supplementary file 1 [file ijms-18-00362-s001.pdf]

Supplementary Materials: Localisation Microscopy of Breast Epithelial ErbB-2 Receptors and Gap Junctions: Trafficking after γ-Irradiation, Neuregulin-1β, and Trastuzumab Application

Götz Pilarczyk, Ines Nesnidal, Manuel Gunkel, Margund Bach, Felix Bestvater and Michael Hausmann

Confocal laser scanning microscopy (CLSM) [1–3] and localization microscopy (LM) [4–7] are called far-field microscopy methods, and are discriminated from near-field approaches [8], indicating that the distance between the specimen observed and the optical system used for imaging are separated by a distance greater than the wavelength of the light used for image generation [9]. In both optical methods, interference processes generate an intermediate image—a process which can be described by a mathematical operation called the Fourier transformation (FT) [10]. In this intermediate image, the fine details of the specimen structure (as well as the optical noise) are projected to the outer regions, while the large specimen structures and the unstructured background (as well as the incident light fraction which did not interact with the specimen) are concentrated around the intermediate image center [11]. By practical means (namely the geometrical maximum diameter possible for the bundle of image rays), the outer areas of the intermediate image are cut off in the imaging pathway [12]. In this way, the capacity of the imaging system to discriminate two specimen points of a certain distance is limited by the spatial restriction of the intermediate image [13]. This is called the optical resolution limit of the particular optical system (i.e., the microscope). A Fourier transformation-based formulation of the imaging system properties is called the object transfer function (OTF). The OTF can be approximated as the realistic form for the idealized microscope's FT. In practice, the image of two sharp specimen points displays both points superposed by an optical smear, with an overlap between the points making it impossible to resolve one point from the other.

In both CLSM and LM, the specimens are the same. A fluorescent dye is specifically accumulated in a non-random fashion, usually by binding the dye molecule to a specific antibody and binding the antibody to the cell's target structure (i.e., the antibody-binding cell protein). In the CLSM case (Figure S1a–c) the fluorophores are excited and emit fluorescence light which is recorded and reflects the cell spatial epitope distribution. During image acquisition, the dye bleaches, and the integral area under the bleaching time course is the determining factor for signal-to-noise ratio and image quality. In the LM case (Figure S1a′–c′) the dye population used for image generation by CLSM recording is bleached and does not contribute to image generation. In contrast, after removal of the main fluorescence molecule population (Figure S1b′), the remaining population develops exotic photocycling and subsequently a characteristic burst-like emission behavior (Figure S1c′). A characteristic difference between CLSM and LM is reflected by the integrals under the fluorescence emission time course curves. While the exponential decaying emission contributes to CLSM image generation, this component is explicitly excluded from LM image generation. In the LM imaging, the isolated bursts are collected over time (Figure S1c′) and re-consolidated in a common matrix with an image of the fluorescence burst localities or a population number weight distance distribution diagram being typical expressions of the data set. A main difference is the emission integral: in CLSM, it is large but decreasing over time of image recording, and in LM, it is much smaller but independent of recording time.

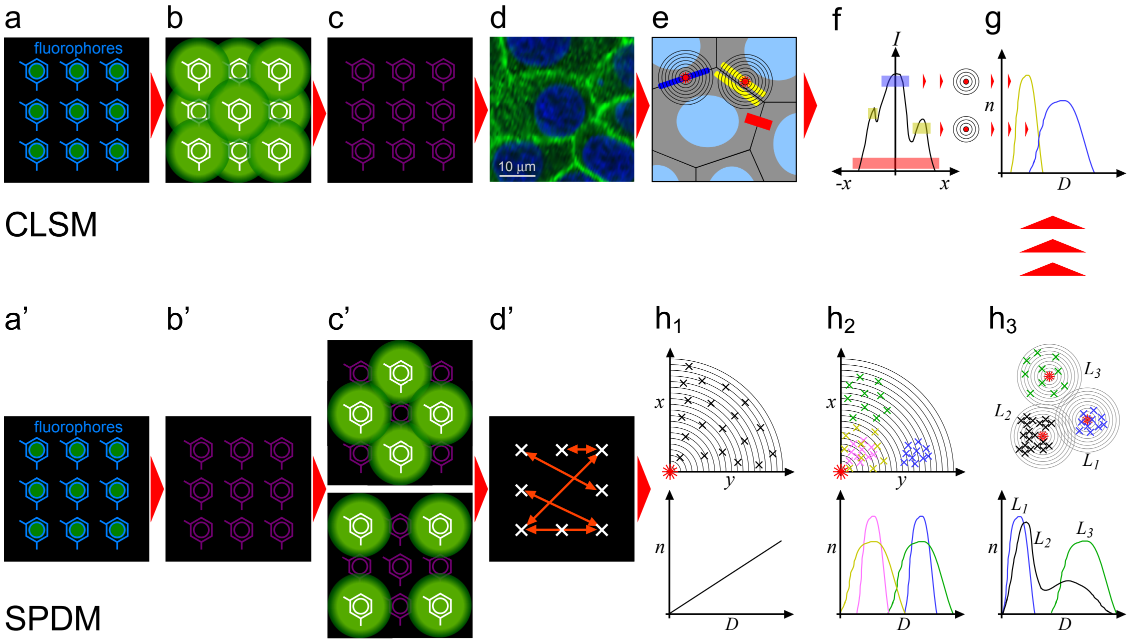

Figure S1. This collection sketches the particular image recording characteristics of confocal laser scanning microscopy (CLSM) and localization microscopy (LM), and shows idealized result situations as generalized examples for data interpretation. (**a**,**a′**) The initial fluorescence dye distribution in the specimen before image recording for CLSM and LM. (**b**,**b′**) Specimen fluorescence emission used (**b**) for CLSM image acquisition and removed (**b′**) by bleaching to prepare for LM image acquisition. (**c**,**c′**) The bleached specimen after (**c**) CLSM image acquisition and during (**c′**) LM image acquisition. The specimen region depleted of conventional fluorescence dye population emits spontaneous short bursts of fluorescence emission. The bursts are isolated in both space and time domains. (**d**,**d′**) CLSM image (**d**) and LM graphical two-dimensional display of the localization matrix (**c′**). (**e**–**g**) Abstract representation of an idealized MCF-7 cell monolayer (**e**) with a region of interest (ROI, red box) and graphical clarification of the cell contact region (blue) and the neighboring cytosol regions (yellow) in adjacent cells. The concentric ring series indicate the mode of LM data-based matrix subset selection for local distance distributions. The line plot intensity profiles (**f**) and the protein molecule density diagrams (**g**) are derived from different cells in comparable condition and appearance. (**h**$_1$–**h**$_3$) A set of typical ErbB or connexin-43 protein accumulations and the respective distance / density distribution after Ripleys K / L-based analysis. For in-depth explanation, see text below.

In CLSM, the intermediate image outer region is cut off by a small circular aperture called the pinhole [14,15]. This procedure is the reason for some special properties characteristic of confocal microscopy. In CLSM imaging, it is possible to reduce the image noise and to increase the image contrast by minimizing the pinhole diameter [16,17]. Opening the pinhole approximates confocal and wide field optical microscope imaging power [18]. Further, the light components from the non-focused specimen areas are excluded by a detector pinhole. This explains the CLSM optical sectioning capacity [19]. A sufficiently small pinhole is capable of suppressing all components of the intermediate image except for the one in the center. This corresponds to the situation where a light spot illuminates the specimen and the image of the light spot is free of the optical smear described above. In conclusion, the pinhole optical filtering makes it possible to minimize a specimen illuminating light spot diameter by suppressing the OTF-induced smear. This increases the optical resolution capacity of CLSM as compared to conventional fluorescence microscopy [18,20]. It must be stated that after pinhole filtering, the specimen image is replaced by the light spot image, and reconstitution of the specimen appearance requires the line-wise and frame-wise scanning of the light spot across the specimen with subsequent image reconstruction in a computer [21].

Figure S1e provides an abstract interpretation of a typical specimen situation, as similarly shown in the CLSM image in Figure S1d. A confluent monolayer of MCF-7 epithelial cells is shown (grey polygons). The cells are reduced to the inter-cellular contacts (black border lines) and schematic nuclei (blue ellipses). The image is subject to a line type (better: slim rectangular box type)-based intensity recording and display. The intensity distribution along the long side of the red box is collected, while each value of the long side is averaged by all corresponding values along the short box edge.The resulting annotation of the intensity values over the local position reflects the local distribution/accumulation of either ErbB receptors or connexin-43 molecules in the region of interest (ROI) selected. The coordinate origin is always centered to the inter-cellular contact line, and the –x to +x extension reflects the distance to the contact line spreading into the cytosol. The round-corner boxes given in blue and yellow in Figure S1e discriminate the close cell contact region from the cytosol near but apart from the immediate cell contacts. Therefore, in the following, LM protein density diagrams and intensity line plots will differ between the membrane immediate region and the cytosolic region of the cells examined.

In Figure S1e, two series of concentric rings with an asterisk in the center give an impression of the LM localities as evaluated by Ripleys K/L type statistics (for detail see below). The diagram in Figure S1g shows that the information given in the LM-derived density distributions correlates with the CLSM-derived intensity information (for detail, see above). The image ROIs of evaluation are not identical due to practical restrictions of the author's local microscopy environment, but in the following, typical regions and situations are identified and compared with each other.

In case of cell specimen imaging, an ensemble of ErbB receptors and gap junction connexons will appear as a continuous accumulation, even if being a group of discrete proteins. Further, spatial alterations in receptor or connexon density restricted to regions smaller than the light spot diameter will not be visible in CLSM recording. In contrast, the thus-averaged specimen structure is a source of information about protein density by the absolute signal level [22]. A high accumulation of ErbB receptors or connexons will result in an increased signal level as compared to a distributed receptor arrangement. Packing density in retrograde trafficking is accessible by CLSM as a cell location-dependent signal height, as given in the principal sketches in Figure S1e–g.

Localization microscopy (LM) does not use an intermediate image filtering, and in this way the method is image information conserving but affected by OTF characteristics. The images are not high image frequency (i.e., noise) filtered, and thus background noise is the main factor limiting the accessible spatial bandwidth [23]. The underlying principle is easy and elegant: the minimum distance limit required for spatial image resolution is circumvented by looking at isolated image points instead of point pairs or ensembles. Even if the image of an isolated specimen point (a fluorescence signal) is smeared out by OTF effects, the center of mass can be localized in space with high precision. A series of individual specimen points each being precisely localized at a time may be used to reconstruct the final image with a spatial resolution being not OTF limited but noise restricted.

Several microscopy methods exploit the principle [6,24–32]. See [33] for a non-fluorescence and [34] for a fluctuation approach, while [35,36] address the need for an increased axial resolution. While microscope hardware, illumination sources, point appearance detection, and image reconstruction are similar, the approaches mainly differ in the generation of spatially-isolated specimen points by the use of different fluorescent dyes [37–39]. The development of burst activity due to exotic photo-cycling behavior is the target of numerous dye chemistry initiatives, leading to a set of fine-tuned organic dyes. The candidates cover a broad range of the light spectrum used in fluorescence microscopy, and recruit from common dye molecule classes as being ATTO, ALEXA, Cy, DIL/DIOC, fluorescent protein, and TAMRA related [40]. In all cases, the Jablonski diagrams for the photo cycles are branched and offer the possibilities for alternative excitation cycles with individual transition probabilities [41]. The ALEXA dyes used in the present approach are capable of following alternative photocycling pathways, and such ways develop fluorescence emission bursts beside their capacity for conventional cw-emission [42,43]. In contrast to fluorescein and rhodamin derivatives where burst

probability is related to dye molecule dissociation induced by pH and ionic strength, cyanine dyes and fluorescent proteins follow an intersystem crossing after cis–trans isomerization (cyanines) or keto–enol tautomerization (FPs) [44–47]. The chemical environment (i.e., the local presence of reactive oxygen species (ROS) and scavengers, nonreactive ions, H^+ and buffers, dye binding Mn^{2+} ions or amino acids side chains) has a tremendous influence on the fluorescence dye burst tendency [48–51]. Vice versa, dye tailoring exploits the structure-dependent burst tendency to design fluorescence dyes with user-controlled burst behavior [52,53]. Again, the requirement for specimen staining is simple: the combination of dye type, dye concentration, and local chemical environment must result in a specimen dye loading where in one image frame the appearing fluorescence signals are isolated by non-fluorescent surroundings. In addition, the focus is on fluorescence photo-cycling modes with low probability, and to discriminate such seldom modes high illumination intensity depopulates photo-cycling modes with high probability. The remaining fluorescence has a burst characteristic of short emission cycle series isolated by long periods of in-excitability (Figure S1a′–d′). In the time domain, the fluorescence bursts are short time signal superpositions on an exponentially decreasing fluorescence background and are mixed with emission noise. Consequently, the image collection can be greatly improved by the application of cameras with high signal dynamics compensating for signal decay during bleach. Furthermore, a camera with a high sensitivity facilitates short acquisition cycles [54,55]. Table S1 illustrates the differences between the confocal (CLSM) and the spatial (LM) microscopy approach.

Data analysis must account for the particular properties of the recorded data sets. The strong dependence of the acquired image stack on dye behavior and the categorical discrete nature of signal appearance independent of the continuous nature of the specimen are determining factors for data processing and interpretation [56,57]. These specimen-related prerequisites are faced by different approaches to derive and formulate the spatial information of discrete specimen points in the acquired data [58]. Over pure localization procedures, the question arises in how far higher orders of signal aggregates are artificial or can contribute to specimen characterization [59,60]. The statistical toolboxes apply Ripleys, Bayesian, and Voronoi-type statistical procedures [61–63] and account for the problem of precision limitations and the extraction of discrete Boolean type information from a continuous grey-scale image set [64,65]. Besides such theoretically orientated considerations, software packages have been designed to collect and analyze microscopy data optimized for beginners [66,67], and also for more advanced applications [68–70]. Additionally, software to estimate the localization power of a certain imaging to evaluation workflow has been developed [71].

A reliable investigation of cell physiologic processes such as membrane receptor and gap junction dynamics requires a measure of cell vitality. Two simple parameters may permit a crude basis for cell state evaluation. A first parameter is the cell size. In epithelial monolayers, an individual cell is mechanically connected to the immediate neighbors, giving the global monolayer a cobblestone appearance with the constituting cells being polygonal. The cell size is not a constant value under this circumstance, because with increasing cell number, epithelial cells tend to increase in thickness and to reduce the area size covered by an individual cell. This is a reason why inside a growing monolayer, cells of different sizes coexist side by side.

As a second parameter, shape and integrity of the cell nucleus can be qualitatively assessed after a conventional nucleic acid stain. The absolute nucleus size is not a reliable value because the monolayer cells are not synchronized with respect to cell division, and thus a distribution of nuclei in different states of chromosome duplication reveal a set of several nucleus sizes. A ratio of nuclear size and cell size is also not a reliable measure, because the cell size is dependent on the cell life time after last division and the local density of the monolayer. In contrast, the cell nucleus appearance can be used as crude measure for cell physiology condition. In a healthy cell being apart from metaphase, there should be only one nucleus without lobes. Fragmentation is an indicator of either apoptosis due to increased cell cycle number or due to mechanical damage by inappropriate preparation. The inner substructure of of the caryoplasm is an effect of the nucleic acid stain used. In our case, the stain (DAPI)

has a slight preference for chromosomal regions with an increased content of adenosine and thymidine bases. The resulting inhomogeneous staining affinity results in a patterned appearance of the nucleus.

Table S1. Comparison of CLSM and LM characteristics with respect to specimen behavior, image recording, and data processing.

Objective	CLSM	LM
Image generation optics	far-field	far-field
Contrast mode	fluorescence, reflectance, etc.	fluorescence
Intermediate image	spatially filtered	unfiltered
Optics resolution power	Abbe/Airy conform	not addressed
Optics ⇒ image	3-D scanner	2-D area detector
Image time series	optional	required
Acquisition time (typical)	4–5 min	4–5 min
Specimen ⇒ image	1-D to 3-D over time	2-D over time
Axial discrimination	1/2–1/4 wide-field	wide-field
Data ⇒ image	scanner coordinates ⋈ intensity	matrix coordinates ⋈ Boolean
Image character	continuous	discrete
Specimen fluorescence	permanent emission	isolated burst
Fluorescence time course	exponential decay	burst level constant
Local signal occurrence	determined	random
Illumination intensity	moderate	high
Image appearance	before bleach	after bleach
Photo-cycles accumulate	signal vanishes	signal intensifies
Absolute noise level	increases over time	decreases over time
Acquisition time extended	signal–noise (SN) ratio decreases	SN ratio increases
SN ratio over time	increases	remains unchanged
Dye bleach ⇒ image formation	destructive	constructive
Fluorescent dyes	photo-stability high	photo-stability low
Jablonski behavior	simple, 1st order exponential	complex, higher orders
Triplet state lifetime	very short	long for burst
Triplet state transition IN	probability high	probability low for burst
Triplet state transition OUT	one way	alternative routes
Chemical environment	importance high	importance low
Embedding media	standardized	object of experimental test
Dye excitation probability	constant, resistant	variable, vulnerable
Dye switching	ignored	required/favored
Dye photo-cyling dynamics	averaged over time	temporally resolved
Image intensity increased	integral bleach curve increased	burst frequency increased
Image continuity	constant over time	increasing over time
Image post-processing	recommended	required
Image converted into values	alternative	required
Post-processing tools	intuitive	intellectual
Image interpretation effort	low	high
Acquisition target amount	concentration	number
Target concentration	intensity/volume	number/area
Further target information	none	distance distribution (*Ripleys K/L*)<br>area size occupied (*Voronoi*)
ErbB/con-43 appearance	bulk<br>continuous population<br>receptor local concentration<br>unstructured population<br>concentration reflects activity<br>packing alters density	individual<br>discrete ensemble<br>local number/clustering<br>several organization levels<br>concentration reflects organization<br>packing alters organization

The supplemental Figure S2 shows the cell nucleus integrity in MCF-7 breast epithelial cells for the four experimental conditions irradiation application, trastuzumab application, neuregulin-1 application, and the unstimulated situation.

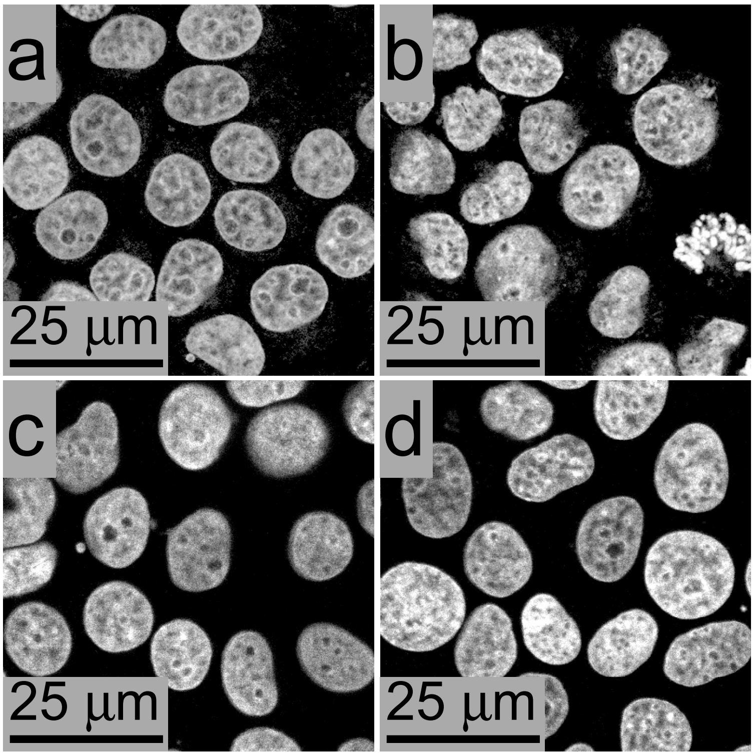

Figure S2. The collection of cell nuclei images clarifies the physiological and mechanical cell integrity of MCF-7 epithelial cells for the four experimental conditions as described above. (**a**) The unstimulated cell. (**b**) Irradiated cells. (**c**) Cells after neuregulin-1 application. (**d**) Cells after trastuzumab application. In each case, the nuclei are of round or slight oval shape. The border between nucleoplasm and cytoplasm is sharp, and there is no nuclei acid stain visible outside the nuclei. Fragmentation or deformations do not appear, indicating that the cells are in healthy conditions and not subjected to an apoptotic process. In (**b**) near the right border, a cell metaphase can be seen. Such cells are not considered for assessment.

References

1. Paddock, S.W.; DeVries, P.; Schatten, G.P. Laser-scanning confocal microscopy and three-dimensional volume rendering of biological structures. *Proc. SPIE* **1990**, *1205*, 20–28.
2. Saunders, R.L. The flying spot microscope. *Can. Med. Assoc. J.* **1952**, *67*, 673–675.
3. Mondal, P.P. Multiple excitation nano-spot generation and confocal detection for far-field microscopy. *Nanoscale* **2010**, *2*, 381–384.
4. Owen, D.M.; Williamson, D.; Magenau, A.; Gaus, K. Optical techniques for imaging membrane domains in live cells (live-cell palm of protein clustering). *Methods Emzymol.* **2012**, *504*, 221–235.
5. Heilemann, M. Fluorescence microscopy beyond the diffraction limit. *J. Biotechnol.* **2010**, *149*, 243–251.
6. Rust, M.J.; Bates, M.; Zhuang, X. Sub-diffraction-limit imaging by stochastic optical reconstruction microscopy (STORM). *Nat. Methods* **2006**, *3*, 793–795.
7. Wegel, E.; Göhler, A.; Lagerholm, B.C.; Wainman, A.; Uphoff, S.; Kaufmann, R.; Dobbie, I.M. Imaging cellular structures in super-resolution with SIM, STED and Localisation Microscopy: A practical comparison. *Sci. Rep.* **2016**, *6*, 1–13.
8. Coello, V.; Bozhevolnyi, S.I. Experimental statistics of near-field intensity distributions at nanostructured surfaces. *J. Microsc.* **2001**, *202*, 136–141.
9. Passon, O.; Grebe-Ellis, J. Note on the classification of super-resolution in far-field microscopy and information theory. *J. Opt. Soc. Am. Ser. A Image Sci. Vis.* **2016**, *33*, B31–B35.
10. Yu, Z.; Prasad, S. High-numerical-aperture microscopy with a rotating point spread function. *J. Opt. Soc. Am. A* **2016**, *33*, B58–B69.

11. Wolf, D.E. The optics of microscope image formation. *Methods Cell Biol.* **2003**, *72*, 11–43.
12. Sluder, G.; Nordberg, J.J. Microscope basics. *Methods Cell Biol.* **2007**, *81*, 1–10.
13. Abbe, E. Note on the proper definition of the amplifying power of a lens or a lens-system. *J. R. Microsc. Soc.* **1884**, *4*, 348–351.
14. Smith, L.W.; Osterberg, H. Diffraction images of circular self-radiant disks. *J. Opt. Soc. Am.* **1961**, *51*, 412–414.
15. Tragardh, J.; Macrae, K.; Travis, C.; Amor, R.; Norris, G.; Wilson, S.H.; Oppo, G.L.; McConnell, G. A simple but precise method for quantitative measurement of the quality of the laser focus in a scanning optical microscope. *J. Microsc.* **2015**, *259*, 66–73.
16. Sheppard, C.J.R.; Gu, M.; Roy, M. Signal to noise ratio in confocal microscope systems. *J. Microsc.* **1992**, *168*, 209–218.
17. Oldenbourg, R.; Terada, H.; Tiberio, R.; Inoue, S. Image sharpness and contrast transfer in coherent confocal microscopy. *J. Microsc.* **1993**, *172*, 31–39.
18. Sandison, D.R.; Piston, D.W.; Williams, R.M.; Webb, W.W. Quantitative comparison of background rejection, signal to noise ratio, and resolution in confocal and full field laser scanning microscopes. *Appl. Opt.* **1995**, *6*, 498–510.
19. Pawley, J.B. Limitations on optical sectioning in live-cell confocal microscopy. *Scanning* **2002**, *21*, 241–246.
20. White, J.G.; Amos, W.B.; Fordham, M. An evaluation of confocal versus conventional imaging of biological structures by fluorescent light. *J. Cell Biol.* **1987**, *105*, 41–48.
21. Nichols, A.J.; Evans, C.L. Video-rate scanning confocal microscopy and microendoscopy. *J. Vis. Exp.* **2011**, *56*, 1–10.
22. Veerdasdonk, J.S.; Lawrimore, J.; Bloom, K. Determining absolute protein numbers by quantitative fluorescence microscopy. *Methods Cell Biol.* **2014**, *123*, 347–365.
23. Shannon, C.E. Communication in the presence of noise. *Proc. IRE* **1949**, *37*, 10–21.
24. Betzig, E.; Patterson, G.H.; Sougrat, R.; Lindwasser, O.W.; Olenych, S.; Bonifacino, J.S.; Davidson, M.W.; Lippincott-Schwartz, J.; Hess, H.F. Imaging intracellular fluorescent proteins at nanometer resolution. *Science* **2006**, *313*, 1642–1645.
25. Hofmann, M.; Eggeling, C.; Jakobs, S.; Hell, S.W. Breaking the diffraction barrier in fluorescence microscopy at low light intensities by using reversibly photoswitchable proteins. *Proc. Natl. Acad. Sci. USA* **2005**, *102*, 17565–17569.
26. Kwoon, J.; Hwang, J.; Park, J.; Han, G.R.; Kim, S.K. RESOLFT nanoscopy with photoswitchable organic fluorophores. *Sci. Rep.* **2015**, *5*, 17804–17812.
27. Oddone, A.; Vilanova, I.V.; Tam, J.; Lakadamyali, M. Super-resolution imaging with stochastic single-molecule localization: Concepts, technical developments, and biological applications. *Microsc. Res. Tech.* **2014**, *77*, 502–509.
28. Jensen, E.; Crossman, D.J. Technical review: Types of imaging-direct STORM. *Proc. Natl. Acad. Sci. USA* **2014**, *297*, 2227–2231.
29. Heilemann, M.; van de Linde, S.; Schüttpelz, M.; Kasper, R.; Seefeldt, B.; Mukherjee, A.; Tinnefeld, P.; Sauer, M. Subdiffraction-resolution fluorescence imaging with conventional fluorescent probes. *Angew. Chem. Int. Ed. Appl. Chem. Int. Ed.* **2008**, *47*, 6172–6176.
30. Hess, S.T.; Girirajan, T.P.K.; Mason, M.D. Ultra-high resolution imaging by fluorescence photoactivation localization microscopy. *Biophys. J.* **2006**, *9*, 4258–4272.
31. Nickerson, A.; Huang, T.; Lin, L.J.; Nan, X. Photoactivated localization microscopy with bimolecular fluorescence complementation (BiFC-PALM) for nanoscale imaging of protein-protein interactions in cells. *PLoS ONE* **2014**, *9*, e100589.
32. Grab, A.L.; Hagmann, M.; Dahint, R.; Cremer, C. Localization microscopy (SPDM) facilitates high precision control of lithographically produced nanostructures. *Micron* **2015**, *68*, 1–7.
33. Zhang, P.; Kim, K.; Lee, S.; Chakkaparani, S.K.; Fang, N.; Kang, S.H. Augmented 3D super-resolution of fluorescence-free nanoparticles using enhanced dark-field illumination based on wavelength-modulation and a least-cubic algorithm. *Sci. Rep.* **2016**, *6*, 32863–32875.
34. Sharonov, A.; Hochstrasser, R.M. Wide-field subdiffraction imaging by accumulated binding of diffusing probes. *Proc. Natl. Acad. Sci. USA* **2006**, *103*, 18911–18916.
35. Huang, F.; Sirinakis, G.; Allgeyer, E.S.; Schroeder, L.K.; Duim, W.C.; Kromann, E.B.; Phan, T.; Rivera-Molina, F.E.; Myers, J.R.; Irnov, I.; et al. Ultra-High Resolution 3D Imaging of Whole Cells. *Cell* **2016**, *166*, 1028–1040.
36. Boehm, U.; Hell, S.W.; Schmidt, R. 4Pi-RESOLFT nanoscopy. *Nat. Commun.* **2016**, *7*, 10504–10512.

37. Chozinski, T.J.; Gagnon, L.A.; Vaughan, J.C. Twinkle, twinkle little star: Photoswitchable fluorophores for super-resolution imaging. *FEBS Lett.* **2014**, *588*, 3603–3612.
38. Xiong, Y.; Rivera-Fuentes, P.; Sezgin, E.; Jentzsch, J.V.; Eggeling, C.; Anderson, H.L. Photoswitchable Spiropyran Dyads for Biological Imaging. *Am. Chem. Soc. Lett.* **2016**, *18*, 3666–3669.
39. Mateos-Gil, P.; Letschert, S.; Doose, S.; Sauer, M. Super-Resolution Imaging of Plasma Membrane Proteins with Click Chemistry. *Front. Cell Dev. Biol.* **2016**, *4*, 98–114.
40. Yang, Z.; Amit, S.; Jing, Q.; Peng, X.; Lee, D.Y.; Hu, R.; Lin, D.; Qu, J.; Kim, J.S. Super-resolution fluorescent materials: An insight into design and bioimaging applications. *Chem. Soc. Rev.* **2016**, *45*, 4651–4567.
41. Zheng, Q.; Juette, M.F.; Jockusch, S.; Wasserman, M.R.; Zhou, Z.; Altman, R.B.; Blanchard, S.C. Ultra-stable organic fluorophores for single-molecule research. *Chem. Soc. Rev.* **2014**, *43*, 1044–1056.
42. De Linde, S.V.; Sauer, M. How to switch a fluorophore: From undesired blinking to controlled photoswitching. *Chem. Soc. Rev.* **2014**, *43*, 1076–1087.
43. Baddeley, D.; Jayasinghe, I.D.; Isuru, D.; Cremer, C.; Cannell, M.B.; Soeller, C. Light-induced dark states of organic fluochromes enable 30 nm resolution imaging in standard media. *Biophys. J. Lett.* **2009**, *96*, L22–L24.
44. Ha, T.; Tinnefeld, P. Photophysics of fluorescent probes for single-molecule biophysics and super-resolution imaging. *Annu. Rev. Phys. Chem.* **2012**, *63*, 595–617.
45. Ke, J.; Wan, Y.; Xia, A.; Li, S.; Gong, F.; Yang, G. Characterization of photoinduced isomerization and intersystem crossing of the cyanine dye Cy3. *J. Phys. Chem. A* **2007**, *111*, 1593–1597.
46. Levitus, M.; Ranjit, S. Cyanine dyes in biophysical research: The photophysics of polymethine fluorescent dyes in biomolecular environments. *Q. Rev. Biophys.* **2011**, *44*, 123–151.
47. Wang, Z.; Chu, S.; Wang, S.; Gong, Q. Cyanine dyes in biophysical research: The photophysics of polymethine fluorescent dyes in biomolecular environments. *J. Chem. Phys.* **2012b**, *137*, 164502–164506.
48. Nani, R.R.; Kelley, J.A.; Ivanic, J.; Schnermann, M.J. Reactive Species Involved in the Regioselective Photooxidation of Heptamethine Cyanines. *Chem. Sci.* **2015**, *6*, 6556–6563.
49. Olivier, N.; Keller, D.; Gönczy, P.; Manley, S. Resolution doubling in 3D-STORM imaging through improved buffers. *PLoS ONE* **2013**, *8*, e69004.
50. Stennett, E.M.S.; Ciuba, M.A.; Levitus, M. Photophysical processes in single molecule organic fluorescent probes. *Chem. Soc. Rev.* **2014**, *43*, 1057–1075.
51. Vogelsang, J.; Steinhauer, C.; Forthmann, C.; Stein, I.H.; Person-Skegro, B.; Cordes, T.; Tinnefeld, P. Make them blink: Probes for super-resolution microscopy. *Chem Phys Chem* **2010**, *11*, 2475–2490.
52. Dempsey, G.T.; Bates, M.; Kowtoniuk, W.E.; Liu, D.R.; Tsien, R.Y.; Zhuang, X. Photoswitching mechanism of cyanine dyes. *J. Am. Chem. Soc. Commun.* **2009**, *131*, 18192–18193.
53. Mahoney, D.P.; Owens, E.A.; Fan, C.; Hsiang, J.C.; Henary, M.M.; Dickson, R.M. Tailoring cyanine dark states for improved optically modulated fluorescence recovery. *J. Phys. Chem. B.* **2015**, *119*, 4637–4643.
54. Almada, P.; Culley, S.; Henriques, R. PALM and STORM: Into large fields and high-throughput microscopy with sCMOS detectors. *Methods* **2015**, *88*, 109–121.
55. Long, F.; Zeng, S.; Huang, Z.L. Localization-based super-resolution microscopy with an sCMOS camera Part II: Experimental methodology for comparing sCMOS with EMCCD cameras. *Optics Express* **2012**, *20*, 17741–17759.
56. MacDonald, L.; Baldini, G.; Storrie, B. Does super resolution fluorescence microscopy obsolete previous microscopic approaches to protein co-localization. *Methods Mol. Biol.* **2015**, *1270*, 255–275.
57. Whelan, D.R.; Bell, T.D.M. Image artifacts in single molecule localization microscopy: Why optimization of sample preparation protocols matters. *Sci. Rep.* **2015**, *5*, 7924–7934.
58. Baddeley, D. Detecting nano-scale protein clustering. *Nat. Methods* **2015**, *12*, 1019–1020.
59. Coltharp, C.; Yang, X.; Xiao, J. Quantitative analysis of single-molecule superresolution images. *Curr. Opin. Struct. Biol.* **2014**, *28*, 112–121.
60. Shivanandan, A.; Unnikrishnan, J.; Radenovic, A. On characterizing protein spatial clusters with correlation approaches. *Sci. Rep.* **2016**, *6*, 31164–31176.
61. Lagache, T.; Lang, G.; Sauvonnet, N.; Olivo-Marin, J.C. Analysis of the spatial organization of molecules with robust statistics. *PLoS ONE* **2013**, *8*, e80914.
62. Kiskowski, M.A.; Hancock, J.F.; Kenworthy, A.K. On the use of Ripleys K-function and its derivatives to analyze domain size. *Biophys. J.* **2009**, *97*, 1095–1103.

63. Tang, Y.; Hendriks, J.; Gensch, T.; Dai, L.; Li, J. Automatic Bayesian single molecule identification for localization microscopy. *Sci. Rep.* **2016**, *6*, 33521–33532.
64. Smith, M.O.; Ball, J.; Holloway, B.B.; Erdelyi, F.; Szabo, G.; Stone, E.; Graham, J.; Lawrence, J.J. Measuring aggregation of events about a mass using spatial point pattern methods. *Spat. Stat.* **2015**, *13*, 76–89.
65. Shivanandan, A.; Unnikrishnan, J.; Radenovic, A. Accounting for limited detection efficiency and localization precision in cluster analysis in single molecule localization microscopy. *PLoS ONE* **2015**, *10*, e0118767.
66. Manley, S.; Gunzenhäuser, J.; Olivier, N. A starter kit for point-localization super-resolution imaging. *Curr. Opin. Chem. Biol.* **2011**, *15*, 813–821.
67. Gustafsson, N.; Culley, S.; Ashdown, G.; Owen, D.M.; Pereira, P.M.; Henriques, R. Fast live-cell conventional fluorophore nanoscopy with ImageJ through super-resolution radial fluctuations. *Nat. Commun.* **2016**, *7*, 12471–12480.
68. Hansson, K.; Jafari-Mamaghani, M.; Krieger, P. RipleyGUI: Software for analyzing spatial patterns in 3D cell distributions. *Front. Neuroinf.* **2013**, *7*, 5–14.
69. Andronov, L.; Lutz, Y.; Vonesch, J.L.; Klaholz, B.P. SharpViSu: Integrated analysis and segmentation of super-resolution microscopy data. *BioInformatics* **2016**, *32*, 2239–2241.
70. Andronov, L.; Orlov, I.; Lutz, Y.; Vonesch, J.L.; Klaholz, B.P. ClusterViSu, a method for clustering of protein complexes by Voronoi tessellation in super-resolution microscopy. *Sci. Rep.* **2016**, *6*, 24084–24093.
71. Girsault, A.; Lukes, T.; Sharipov, A.; Geissbuehler, S.; Leutenegger, M.; Vandenberg, W.; Dedecker, P.; Hofkens, J.; Lasser, T. SOFI Simulation Tool: A Software Package for Simulating and Testing Super-Resolution Optical Fluctuation Imaging. *PLoS ONE* **2016**, *11*, e0161602.
